# Rapid Diagnostics for Distinguishing Bacterial and Viral Infections: A Review of Technologies, Clinical Utility, and Stewardship Implications

**DOI:** 10.3390/bios16090499

**Published:** 2026-09-06

**Authors:** Mohammad Javanmard, Rohan Vellanki, Ali Fardoost, Mehdi Javanmard

**Affiliations:** Department of Electrical and Computer Engineering, Rutgers University, Piscataway, NJ 08854, USA; mo.javanmard.25@gmail.com (M.J.); rohan.vellanki5@gmail.com (R.V.); af1069@scarletmail.rutgers.edu (A.F.)

**Keywords:** antimicrobial resistance (AMR), bacterial and viral infection, rapid diagnostics, host-response diagnostics, point-of-care testing (POCT), biosensors, biomarkers, antimicrobial stewardship

## Abstract

Antimicrobial resistance (AMR) is a growing global health threat driven in part by inappropriate and unnecessary antibiotic use resulting from diagnostic uncertainty at the point of care. In outpatient and acute-care settings, clinicians are often unable to rapidly distinguish between viral and bacterial infections, leading to empiric antibiotic prescribing that contributes to the emergence and spread of resistant pathogens. This review examines current and emerging rapid diagnostic technologies for differentiating bacterial and viral infections, including molecular assays, rapid antigen tests, biomarker-based diagnostics, host-response platforms, hematologic methods, and artificial intelligence-based decision-support systems. These technologies are evaluated based on diagnostic accuracy, turnaround time, cost, accessibility, and clinical actionability within real-world healthcare settings. Although several emerging and point-of-care (POC) technologies can provide results within approximately 6–15 min, their ability to consistently align with the timing, workflow, and clinical decision-making requirements of frontline outpatient and emergency-care settings remains variable and incompletely established. Future progress in antimicrobial stewardship will depend on developing rapid, clinically actionable diagnostic systems that integrate seamlessly into patient care and reduce unnecessary antibiotic use.

## 1. Introduction

The public health dangers of antimicrobial resistance (AMR) continue to increase worldwide. A global analysis estimated that approximately 1.27 million deaths were directly attributable to bacterial AMR in 2019, with an additional 4.95 million deaths associated with drug-resistant infections [1]. Projections suggest that annual AMR-related mortality could approach 10 million deaths by 2050 if current trends persist [2]. Additionally, AMR presents major economic risks. Reports from the World Bank and World Health Organization indicate that drug-resistant infections may substantially increase healthcare expenditures while contributing to significant losses in global economic productivity and gross domestic product [3,4].

AMR is driven by natural selection, in which excessive antibiotic use suppresses vulnerable bacterial populations while resistant strains survive and multiply [5,6]. Resistance develops through both genetic mutation and horizontal gene transfer, with key clinical mechanisms including enzymatic drug inactivation (e.g., β-lactamase production), reduced membrane permeability, active efflux, and modification of antibiotic targets [7,8]. These processes have expanded rapidly in response to widespread antibiotic use, contributing to the global emergence and spread of resistant pathogens [1,5].

A major contributor to AMR in clinical practice is diagnostic uncertainty during the initial patient encounter. In outpatient and emergency-care settings, clinicians are frequently unable to rapidly and reliably distinguish between viral and bacterial infections [9,10]. Patients commonly present with nonspecific symptoms such as fever, cough, sore throat, and fatigue, which are shared across both viral and bacterial infections and lack sufficient discriminatory value when considered in isolation. Consequently, physicians must often rely on clinical judgment, risk stratification, and prior experience to guide initial management, frequently resulting in empiric antibiotic prescribing when bacterial infection cannot be confidently excluded [9,10]. In the United States, approximately 30% of outpatient antibiotic prescriptions are estimated to be unnecessary, reflecting the challenges associated with making treatment decisions in the absence of definitive diagnostic information [11,12]. This pattern of empiric prescribing contributes directly to the selective pressures that drive the development and spread of resistant organisms [5,8].

The consequences of unnecessary antibiotic exposure extend beyond long-term resistance and include immediate patient-level harms. Antibiotics provide no benefit in viral infections and may expose patients to allergic reactions, gastrointestinal disturbances, and disruption of the normal microbiome. Surveillance studies have also demonstrated that antibiotic-associated adverse events account for a substantial number of emergency department visits annually, most commonly due to allergic reactions and other drug-related complications [13]. Thus, even modest reductions in unnecessary antibiotic use may generate meaningful improvements in patient safety while reducing selective pressure for AMR.

This diagnostic challenge is compounded by the limitations of existing clinical workflows. Definitive microbiological methods, such as bacterial culture, typically require 24–72 h to yield results, with additional time often needed for organism identification and antimicrobial susceptibility testing [14,15]. Although molecular diagnostics such as polymerase chain reaction (PCR) have significantly improved analytical accuracy and reduced turnaround times compared with culture, they may still require 30–60 min or longer and are not always accessible in outpatient or emergency-care settings [10,16]. Faster modalities, including rapid antigen tests and individual biomarker assays, can provide results within clinically relevant timeframes but may lack sufficient sensitivity or specificity to independently guide treatment decisions [17,18]. Consequently, clinicians are frequently required to initiate treatment before definitive diagnostic information becomes available.

Prescribing behavior is further influenced by systemic and behavioral factors, including time pressure in emergency and urgent-care settings, perceived patient expectations, and concern about missing a serious bacterial infection [11,19]. These factors can promote precautionary antibiotic use even when the underlying etiology is likely viral. Therefore, the inability to rapidly and reliably distinguish between bacterial and viral infections at the point of care represents a central challenge in infectious disease management [10,20]. The clinical value of a diagnostic technology is consequently determined not only by its analytical performance, but also by whether it can provide actionable information within the narrow window in which treatment decisions are made [10,21]. Technologies that generate highly accurate results only after prescribing decisions have already occurred may have limited practical impact on antimicrobial stewardship.

Although numerous previous reviews have examined bacterial-versus-viral differentiation, much of this literature has focused on specific clinical syndromes, particularly respiratory tract infections; individual biomarkers; or the analytical performance of host-response signatures [22,23,24]. Comparatively less attention has been given to the broader question of how different diagnostic modalities perform within the real-time clinical decision window, particularly in diagnostically challenging non-respiratory presentations such as undifferentiated sepsis. Furthermore, diagnostic accuracy does not necessarily translate into changes in antimicrobial prescribing, and evidence linking diagnostic technologies to actual clinical decision-making remains heterogeneous.

Therefore, the present review adopts a clinical-actionability and antimicrobial-stewardship-oriented framework rather than focusing solely on diagnostic accuracy. We compare molecular, antigen-based, biomarker, host-response, hematologic, and AI-assisted approaches according to not only analytical performance, but also turnaround time, accessibility, workflow compatibility, and potential or demonstrated influence on antibiotic decision-making. Particular emphasis is placed on host-response and multimodal approaches that may be applicable beyond respiratory infections, including systemic and undifferentiated infectious syndromes such as sepsis. This framework is intended to identify the remaining gap between successful bacterial-versus-viral classification and demonstrated improvement in real-world antimicrobial decision-making.

An ideal diagnostic solution would therefore provide rapid, accurate, and clinically actionable results during the patient encounter, enabling physicians to more confidently differentiate between viral and bacterial infections in real time. Such tools could support more appropriate antibiotic use, reduce unnecessary exposure, and improve both patient safety and antimicrobial stewardship. Achieving alignment between diagnostic timing, diagnostic performance, and clinical decision-making remains a defining challenge in the development of next-generation infectious disease diagnostics.

Clinical use cases considered in this review should be distinguished according to the question being addressed. Rapid diagnostic technologies may be used for (i) pathogen detection, where the objective is to identify a predefined bacterial or viral agent; (ii) etiologic classification, where the objective is to determine whether an undifferentiated illness is more likely bacterial or viral/non-bacterial; or (iii) severity and sepsis assessment, where the objective is to identify patients at increased risk of severe infection or systemic deterioration. These objectives are complementary rather than interchangeable. For example, a positive pathogen-specific PCR establishes the presence of a target organism, whereas a host-response assay estimates the likelihood of bacterial versus viral infection, and an AI-based sepsis model may estimate clinical deterioration risk without identifying the causative pathogen. Throughout this review, these distinctions are maintained when considering diagnostic performance, clinical actionability, and potential antimicrobial-stewardship impact.

## 2. Methods

This study was conducted as a structured narrative review. Literature searches were performed in PubMed/MEDLINE, Scopus, Web of Science Core Collection, and Embase, with the final search conducted in July 2026. The search strategy combined four main concepts: (1) bacterial and viral infection or etiologic differentiation, (2) rapid/point-of-care diagnostics, (3) biosensor, biomarker, molecular, antigen, host-response, hematologic, or computational approaches, and (4) antimicrobial stewardship or antibiotic decision-making. Representative search terms included combinations of bacterial infection, viral infection, bacterial versus viral, etiologic diagnosis, rapid diagnostic, point-of-care, POC, molecular diagnostic, PCR, antigen test, biomarker, host response, biosensor, microfluidic, hematology, artificial intelligence, machine learning, antimicrobial stewardship, antibiotic prescribing, and antibiotic use. Database-specific syntax and controlled vocabulary were adapted to each database.

Studies were considered eligible when they addressed rapid or near-patient approaches relevant to infectious-disease assessment and provided information on at least one of the following: diagnostic or analytical performance, bacterial-versus-viral differentiation, pathogen detection, host-response characterization, clinical implementation, turnaround time, or antimicrobial decision-making. Studies describing relevant biosensor, microfluidic, sample-to-answer, or other device-level approaches were also considered when they provided information relevant to rapid infectious-disease testing. English-language publications were prioritized. Studies focused exclusively on unrelated noninfectious applications, technologies without relevance to infectious-disease diagnosis or clinical decision support, or reports lacking information relevant to the objectives of this review were excluded. Reference lists of relevant reviews and primary studies were screened to identify additional pertinent publications.

The review was intentionally structured as a narrative synthesis rather than a systematic review or meta-analysis. Consequently, studies were not selected to generate pooled estimates of diagnostic accuracy. Instead, representative technologies were identified according to their clinical relevance to bacterial-versus-viral diagnostic uncertainty, technological maturity, point-of-care or rapid-testing potential, relevance to antimicrobial stewardship, and availability of traceable clinical or analytical evidence. Particular emphasis was placed on technologies that illustrate distinct diagnostic strategies, including pathogen-directed molecular and antigen testing, host-response and biomarker approaches, hematologic measurements, and computational decision-support systems.

In this review , studies were selected using an additional evidence-availability criterion. Preference was given to publications that provided traceable quantitative information for one or more parameters included in the table, including clinical sensitivity/specificity or PPA/NPA, reference standard, limit of detection where applicable, sample-to-result time, and clinical or antimicrobial-stewardship evidence. When multiple studies were available for the same platform, independent prospective or multicenter clinical evaluations were prioritized for clinical diagnostic performance, while manufacturer instructions for use and regulatory documentation were used primarily for intended use, specimen requirements, analytical characteristics, operating time, and regulatory status. The selected studies therefore represent representative evidence for each technology rather than a statistically comparable set of studies.

Because the technologies differ in clinical indication, patient population, specimen type, target analyte, reference standard, and diagnostic task, quantitative values in this study are presented in their original study context and are not intended to constitute a direct head-to-head ranking of diagnostic accuracy. Where quantitative information was unavailable, not applicable, or insufficiently comparable, it was reported as not reported (NR) or not applicable (N/A), rather than inferred from other studies. For multiplex platforms, analyte-specific performance was used where available, and overall panel-level performance was not interpreted as representative of every individual target.

Manufacturer instructions for use, technical documentation, and publicly available regulatory records were reviewed separately from peer-reviewed clinical evidence. These sources were used primarily to establish intended use, specimen type and volume, analytical characteristics, operating or sample-to-result time, instrumentation requirements, and regulatory status. Document title, version or revision information where available, issuing organization, and access date were recorded for manufacturer and regulatory sources. Independent prospective, multicenter, randomized, and implementation studies were given greater weight when evaluating clinical diagnostic performance, antibiotic prescribing, and patient outcomes. No formal systematic-review risk-of-bias assessment or meta-analysis was performed.

## 3. Major Categories of Rapid Diagnostics

Rapid diagnostic technologies can be categorized according to the clinical question they are designed to answer and their ability to resolve bacterial-versus-viral diagnostic uncertainty. From the perspective of antimicrobial stewardship, diagnostic approaches may directly identify bacterial or viral pathogens, indirectly characterize the likelihood of bacterial versus viral infection through biomarkers or host-response patterns, or integrate multiple clinical measurements to support treatment decisions. Accordingly, current approaches can be grouped into molecular tests, rapid antigen assays, biomarker-based diagnostics, host-response platforms, hematologic approaches, and artificial intelligence (AI)-based decision-support systems. These categories differ not only in analytical performance, but also in their ability to distinguish bacterial from viral infection, provide results within the clinical decision window, and generate information that can support appropriate antibiotic initiation or avoidance. The following sections therefore consider each technology not only in terms of its technical capabilities, but also in relation to its potential clinical actionability and relevance to antimicrobial stewardship.

### 3.1. Molecular Tests

Molecular diagnostic platforms, including real-time PCR, reverse-transcription PCR, nested multiplex PCR, and isothermal nucleic acid amplification tests (NAATs), detect pathogen-specific nucleic acid sequences and therefore provide a pathogen-directed approach to bacterial-versus-viral differentiation. By amplifying small amounts of microbial DNA or RNA, these assays can achieve high analytical sensitivity and specificity while substantially reducing turnaround time compared with conventional culture [9,16]. From an antimicrobial-stewardship perspective, their principal value is the potential to replace empiric treatment based solely on clinical uncertainty with microbiological information available early enough to influence antibiotic decisions. However, the extent to which an individual molecular assay contributes to bacterial-versus-viral differentiation depends on its target coverage and the clinical probability of the organism being tested.

Molecular amplification platforms provide high analytical sensitivity through sequence-specific nucleic-acid recognition and enzymatic amplification. Systems such as GeneXpert use automated sample preparation, nucleic-acid extraction, amplification, and fluorescence-based detection within disposable cartridges, reducing hands-on processing and supporting sample-to-answer operation. For example, Xpert Xpress Strep A (“Strep A” referring to Group A Streptococcus, i.e., *Streptococcus pyogenes*) (Cepheid, Sunnyvale, CA, USA) reports analytical detection limits of approximately 9–18 CFU/mL for evaluated *S. pyogenes* strains [25]. BioFire FilmArray RP2.1 (BioFire Diagnostics, Salt Lake City, UT, USA) similarly integrates sample preparation, multiplex nucleic-acid amplification, and fluorescence detection within a closed cartridge and can simultaneously interrogate numerous respiratory pathogens; however, its analytical detection limit is target-dependent rather than represented by a single value [26]. These platforms generally require dedicated readers and cartridges and therefore offer high reproducibility and mature manufacturability at the expense of instrument cost and consumable expense. Internal process controls and standardized cartridge workflows provide important quality-control advantages, whereas specimen matrix effects, amplification inhibitors, contamination, and target selection remain relevant considerations. Multiplex molecular systems provide substantially broader etiologic information than single-pathogen assays, but detection of nucleic acid does not necessarily establish causation, particularly when colonization or residual nucleic acid is possible.

POC and sample-to-answer NAAT platforms address an important limitation of conventional molecular diagnostics by integrating sample preparation, nucleic acid extraction, amplification, and detection into automated workflows. These systems can provide highly specific pathogen identification within minutes to approximately one hour, making molecular information more compatible with the clinical decision window [27,28,29,30,31]. Importantly, the underlying sample-to-answer technology is not restricted to a particular organism. The same general platform architecture can be configured for different bacterial or viral targets, allowing molecular testing strategies to be adapted to local epidemiology, clinical syndromes, and pathogen prevalence. Nevertheless, a single-target assay can only answer a narrow diagnostic question. A negative result for one bacterial pathogen does not establish a viral etiology, and therefore targeted molecular testing is most useful when clinical findings, epidemiological information, or local pathogen prevalence create a sufficiently strong pre-test suspicion for the specific organism.

From a device-engineering perspective, sample-to-answer molecular diagnostics depend on integration of several otherwise separate operations, including specimen metering, cell or viral-particle lysis, nucleic-acid extraction, purification, amplification, and signal detection [32]. Microfluidic architectures can perform these steps within disposable cartridges using capillary forces, pressure-driven flow, centrifugal actuation, valves, or other fluid-control strategies [33]. Such integration can reduce hands-on processing and contamination risk while shortening the time between sample collection and result generation. However, increasing automation does not eliminate engineering challenges. Complex specimens may require efficient lysis and inhibitor removal, while multiplex amplification introduces competition between targets and requires careful optimization of primer and probe concentrations [34]. Thus, sample-to-answer integration should be evaluated not only according to whether all assay steps are physically incorporated into a cartridge, but also according to whether the integrated workflow provides sufficiently reliable results within the clinical decision window.

This limitation provides an important rationale for multiplex molecular testing. Syndromic multiplex panels can simultaneously evaluate numerous bacterial and viral targets from a single clinical specimen, thereby providing broader etiologic information within a clinically relevant timeframe. The BioFire FilmArray Respiratory Panel 2.1, for example, uses a closed-pouch multiplex workflow to detect a broad range of respiratory pathogens, including viruses and atypical bacteria, with a reported run time of approximately 45 min and high overall analytical performance [35,36]. Unlike a single-pathogen assay, such a panel can provide simultaneous evidence for alternative infectious etiologies when the clinical presentation does not clearly indicate whether the illness is bacterial or viral. This broader coverage may be particularly valuable for antimicrobial stewardship because identification of a viral etiology may support avoidance of unnecessary antibiotics, while identification of a bacterial pathogen may support targeted treatment rather than broad empiric therapy.

However, multiplex testing should not be equated automatically with improved antimicrobial stewardship. Detection of microbial nucleic acid does not necessarily establish that the organism is responsible for the patient’s symptoms. Viral nucleic acid may persist after the acute phase of infection, while bacterial detection from respiratory specimens may represent colonization rather than invasive disease. Co-detection of multiple organisms can also complicate interpretation and may increase rather than decrease diagnostic uncertainty if clinical context is not considered. In addition, multiplex panels may involve substantially greater costs and infrastructure requirements than targeted assays and may not be necessary when a specific pathogen is strongly suspected from clinical or epidemiological information. Thus, the optimal molecular strategy is context-dependent: targeted NAATs can provide rapid, high-confidence answers when the suspected pathogen is well defined, whereas multiplex panels offer broader coverage when bacterial-versus-viral etiology is uncertain.

Targeted Strep A testing provides a useful example of the former approach. *Streptococcus pyogenes* is an important bacterial cause of acute pharyngitis, but most cases of acute pharyngitis are viral, making unnecessary antibiotic prescribing an important stewardship concern in this clinical setting. Rapid molecular testing can therefore be clinically valuable when bacterial pharyngitis is suspected but cannot be reliably established from symptoms alone. The Cepheid Xpert Xpress Strep A assay detects *S. pyogenes* DNA using an automated cartridge-based real-time PCR workflow and reports negative results in approximately 24 min and strongly positive results as early as 18 min [27,28]. Similarly, the Roche cobas Liat Strep A (Roche Diagnostics, Rotkreuz, Switzerland) assay provides approximately 15-min results with reported sensitivity of 97.7% and specificity of 93.3% compared with reference culture [29,30], while the Abbott ID NOW Strep A 2 (Abbott Diagnostics, Scarborough, ME, USA) uses isothermal amplification and can provide results in 6 min or less, with reported sensitivity of 98.5% and specificity of 93.4% against culture [31]. These examples demonstrate how the same rapid molecular principles can provide clinically actionable bacterial confirmation during a patient encounter.

**Figure 1:**
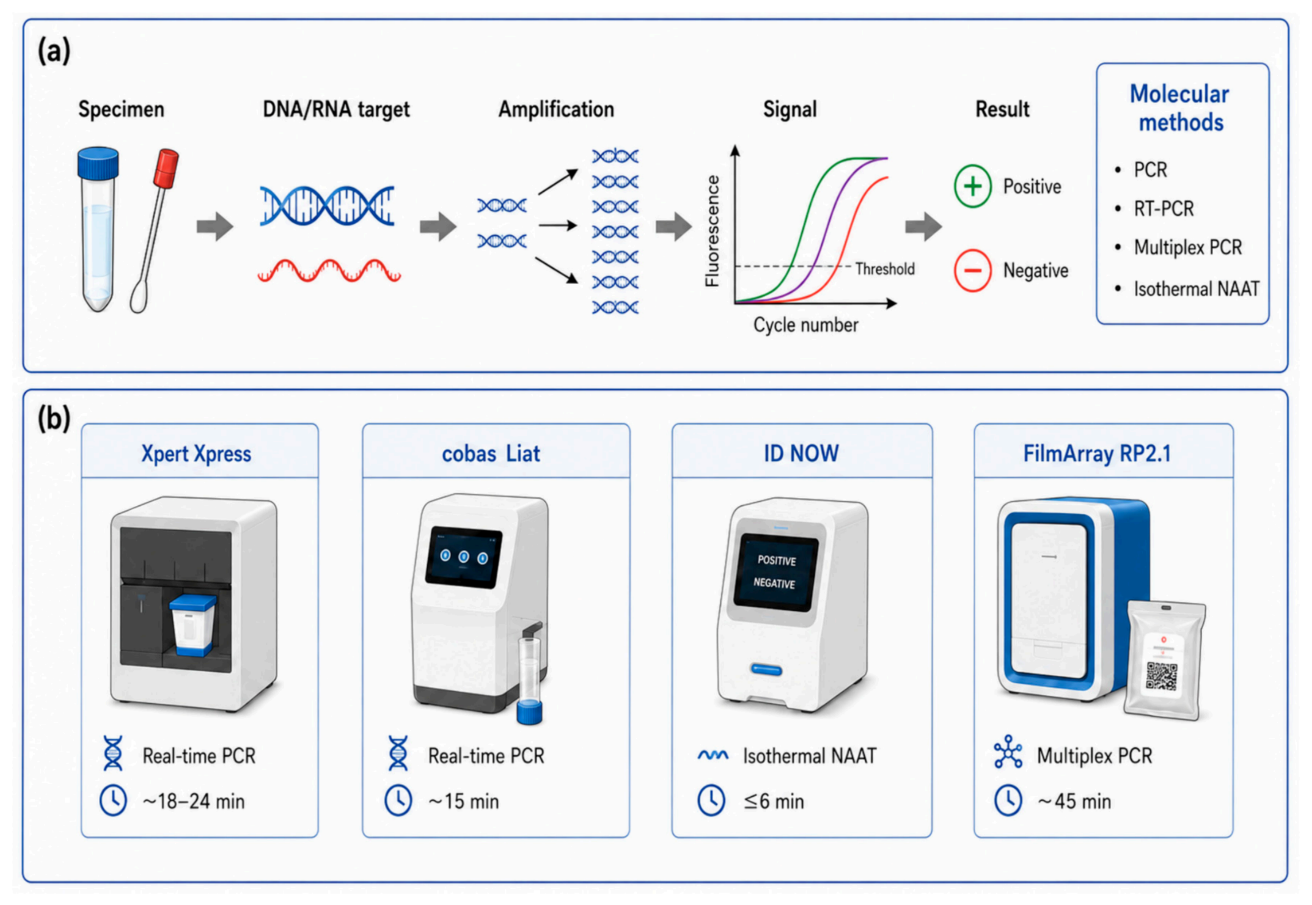
Representative molecular diagnostic approaches for infectious disease diagnosis. (**a**) Core molecular testing workflow, beginning with patient specimen collection, followed by pathogen DNA or RNA target recognition, amplification, signal detection, and positive or negative result interpretation. (**b**) Examples of near-patient molecular platforms, including real-time PCR, isothermal NAAT, and multiplex PCR systems, used for rapid pathogen detection.

The significance of these targeted assays, however, lies less in Strep A itself than in the diagnostic model they represent. Rapid pathogen-specific NAATs can be valuable when clinical or epidemiological information provides a focused diagnostic hypothesis, but they cannot provide comprehensive bacterial-versus-viral classification when the differential diagnosis is broad. Their greatest AMR relevance therefore arises when they are appropriately matched to the clinical syndrome and used within a workflow in which results can influence antibiotic prescribing. Multiplex molecular platforms extend this principle by broadening the range of pathogens evaluated within a single encounter, although their stewardship benefit ultimately depends on appropriate interpretation, timely availability, and evidence that the resulting information changes antimicrobial decisions. Representative molecular diagnostic workflows and near-patient molecular platforms are summarized in Figure 1.

### 3.2. Rapid Antigen Tests

Rapid antigen detection tests (RADTs) are widely used because of their low cost, operational simplicity, and ability to deliver results within minutes at the point of care. Unlike molecular assays that detect nucleic acid, RADTs identify pathogen-specific proteins, most commonly surface antigens or nucleocapsid proteins, through antibody-based immunoassay systems. The majority of commercially available RADTs use lateral-flow immunochromatographic technology, in which a patient sample migrates across a membrane containing immobilized antibodies directed against specific microbial antigens. If the target antigen is present, antigen–antibody complexes accumulate at a detection zone and generate a visible signal, typically through colloidal gold nanoparticles or fluorescent labels. Because these assays do not require nucleic acid amplification or complex instrumentation, they can be deployed rapidly in outpatient clinics, pharmacies, urgent care centers, schools, and emergency departments [17,37].

From an antimicrobial-stewardship perspective, the role of RADTs requires an important distinction between confirming a specific viral infection and excluding bacterial infection. A positive viral antigen result can provide rapid etiologic information that supports a viral diagnosis and may reduce the perceived need for empiric antibiotics when the clinical presentation is otherwise consistent with a viral illness. However, a negative viral RADT does not establish a bacterial etiology, and even a positive viral result does not completely exclude bacterial coinfection. Therefore, viral RADTs should be considered complementary components of bacterial-versus-viral assessment rather than standalone rule-out tests for bacterial disease. Their clinical value is greatest when test results are available during the treatment decision window and are interpreted together with clinical findings, patient risk factors, epidemiological information, and bacterial testing when clinically indicated.

One of the most widely used RADT applications is influenza detection. The BD Veritor System for Rapid Detection of Flu A + B (Becton, Dickinson and Company, Franklin Lakes, NJ, USA) uses a chromatographic immunoassay combined with an automated optical reader to detect influenza nucleoprotein antigens from respiratory swabs [38]. The assay produces results in approximately 10–15 min, while rapid influenza diagnostic tests more broadly have reported variable sensitivity depending on viral load, specimen quality, and timing of presentation [17,38]. Specificity is generally high, often exceeding sensitivity across rapid influenza antigen tests [17]. Unlike visually interpreted lateral-flow assays, the use of a digital analyzer improves signal interpretation and reduces user-dependent variability. However, sensitivity remains substantially lower than RT-PCR, particularly during early or low-viral-load infection [17]. Thus, influenza RADTs can provide clinically useful positive viral information rapidly, but a negative result should not independently be interpreted as evidence of bacterial infection.

SARS-CoV-2 antigen assays became widely adopted during the COVID-19 pandemic because they enabled decentralized mass testing without laboratory infrastructure. The Abbott BinaxNOW (Abbott Diagnostics, Scarborough, ME, USA) COVID-19 Ag Card uses a lateral-flow immunoassay format targeting the SARS-CoV-2 nucleocapsid protein [39]. In this system, the extracted nasal swab antigen binds to labeled antibodies and migrates across a membrane, where immobilized capture antibodies generate a visible test line. Reported specificity is consistently high, while sensitivity varies substantially depending on viral load, symptom duration, and patient population [37,39,40,41,42]. Studies have reported higher performance among symptomatic individuals during peak viral shedding, while lower sensitivity in some settings increases the risk of false-negative results [37]. Nevertheless, rapid turnaround times of approximately 15 min enabled widespread use in community screening and infection-control programs [39]. For antimicrobial stewardship, the relevance of such viral testing is therefore not that it directly demonstrates the absence of bacterial infection, but that a timely positive result can provide an alternative etiologic explanation for symptoms and potentially reduce unnecessary antibacterial treatment when the clinical context supports a viral diagnosis.

Rapid antigen detection is also commonly used for Group A Streptococcus (GAS) pharyngitis. The Quidel QuickVue Dipstick Strep A Test (Quidel Corporation, San Diego, CA, USA) detects GAS carbohydrate antigen using enzyme-linked immunochromatographic technology [43]. In this assay, extracted streptococcal antigen reacts with labeled antibodies embedded within the test strip to produce a visible colorimetric result. Reported specificity is generally high, while sensitivity is lower and more variable than molecular testing or reference culture [27,43]. Because false-negative results remain clinically significant, especially in pediatric populations, negative RADT results may require confirmatory culture or molecular testing depending on clinical context and local guidelines [27,43]. In contrast to viral RADTs, targeted bacterial antigen testing can provide direct evidence supporting antibacterial treatment; however, like other pathogen-specific tests, it does not comprehensively characterize the broader bacterial-versus-viral differential.

Rapid antigen tests use pathogen-specific antibodies as the biorecognition elements and generally rely on lateral-flow immunochromatography, with visual or reader-assisted optical interpretation. Their principal device-level advantage is minimal sample preparation and small specimen requirements, allowing simple decentralized workflows without nucleic-acid extraction or amplification. However, their analytical sensitivity is substantially dependent on antigen abundance and specimen quality. For example, reported analytical detection limits for rapid influenza antigen tests vary widely among platforms and viral strains, illustrating that a single LOD cannot be generalized across the technology class [44]. BinaxNOW and BD Veritor are mature, scalable lateral-flow formats, whereas reader-assisted systems can reduce subjective visual interpretation. QuickVue Strep A similarly provides rapid pathogen-specific antigen detection but has a substantially higher analytical detection threshold than molecular Strep A assays, illustrating the trade-off between simplicity and analytical sensitivity [45]. These assays generally require procedural controls rather than complex calibration, but matrix effects, specimen quality, low pathogen burden, and antigenic variation can influence performance. Multiplexing is technically possible, although many established lateral-flow products remain single- or limited-target assays.

Evidence that rapid viral testing reduces antibiotic use in real-world practice is encouraging but heterogeneous. Importantly, evidence of clinical impact should not be generalized across rapid viral-testing platforms or clinical settings. Recent randomized evidence synthesized across molecular point-of-care respiratory testing found little to no overall reduction in antibiotic use or treatment duration, despite an improvement in prescribing appropriateness [46]. This suggests that identifying a viral pathogen can improve diagnostic certainty without necessarily changing the initial antibiotic decision. The stewardship effect is therefore likely to depend on the clinical context, timing of testing, pretest probability of bacterial disease, availability of follow-up or confirmatory testing, and whether clinicians are supported by an explicit treatment pathway.

Several studies have demonstrated that providing rapid influenza or respiratory-virus results to clinicians can reduce antibiotic prescribing or antibiotic exposure in selected settings. For example, randomized studies in pediatric emergency settings have reported reduced antibiotic use when clinicians received rapid influenza results, while a randomized outpatient study found lower initial antibiotic prescribing when rapid multiplex viral PCR results were available during the initial visit rather than being delayed [47,48]. However, this effect is not universal. A large randomized clinical trial of rapid respiratory pathogen testing in children with influenza-like illness found no reduction in antibiotic prescribing, despite providing results within approximately 45 min [49]. Similarly, implementation studies have shown that rapid respiratory panels may shorten antibiotic duration without necessarily reducing the proportion of patients initially prescribed antibiotics [50]. These findings demonstrate that rapid viral identification alone is insufficient to guarantee stewardship benefit; clinician interpretation, prescribing norms, diagnostic confidence, and workflow integration strongly influence whether test results translate into changed antibiotic behavior.

Despite their operational advantages, RADTs possess important limitations for antimicrobial stewardship. Their lower analytical sensitivity compared with molecular assays increases the likelihood of false-negative results, particularly during early infection or low pathogen burden. In addition, RADTs remain pathogen-specific and therefore do not directly answer the broader clinical question of whether a patient’s illness is bacterial or viral overall. A negative influenza or SARS-CoV-2 antigen test does not exclude alternative viral infection, while positive bacterial antigen detection may not fully characterize disease severity or coinfection risk. Consequently, although RADTs improve diagnostic accessibility and workflow compatibility, their contribution to reducing unnecessary antibiotic prescribing remains highly dependent on clinical interpretation and confirmatory testing strategies [17,37].

### 3.3. Biomarkers

Biomarker-based diagnostics provide an indirect approach to bacterial-versus-viral differentiation by measuring host or inflammatory responses rather than directly detecting the causative pathogen. Commonly evaluated biomarkers include C-reactive protein (CRP) and procalcitonin (PCT), which can provide information about the likelihood or severity of bacterial infection but generally cannot establish microbial etiology independently. Their clinical value therefore lies primarily in reducing diagnostic uncertainty and supporting, rather than replacing, clinical assessment. For antimicrobial stewardship, this distinction is important because a biomarker that indicates a lower probability of bacterial infection may support withholding or discontinuing antibiotics, whereas an elevated inflammatory marker alone does not necessarily justify antibacterial treatment [18,51,52].

**Figure 2:**
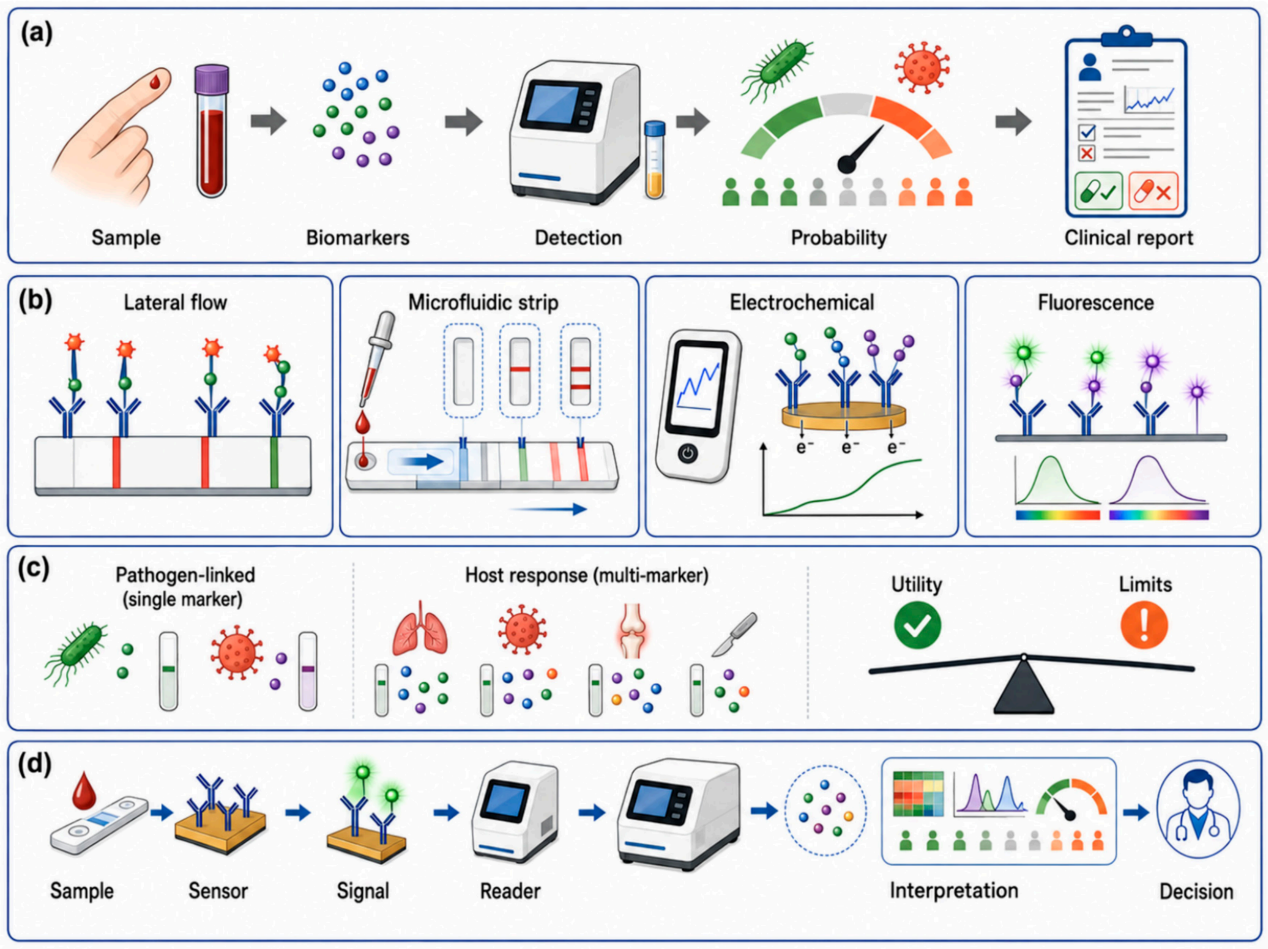
Biomarker-based diagnostic approaches for assessing bacterial-versus-viral infection. All panels are conceptual representations of general diagnostic principles and do not represent the complete workflow or output of a single commercial platform. (**a**) General workflow from patient sample collection, including blood or fingerstick sampling, through biomarker measurement and probabilistic interpretation. (**b**) Representative biomarker detection technologies, including lateral-flow immunoassays, microfluidic platforms, electrochemical biosensors, and fluorescence-based detection. (**c**) Conceptual comparison of single-biomarker and multi-biomarker host-response strategies. Single inflammatory biomarkers such as CRP and PCT provide indirect information about the probability of bacterial infection, whereas multi-marker approaches can integrate complementary host-response signals to improve etiologic discrimination. (**d**) Conceptual integrated workflow illustrating how biomarker measurements may be processed, interpreted, and incorporated with clinical information to support clinical decision-making. The relationships shown are based on the host-response and biomarker literature discussed in the manuscript [51,52,53,54,55,56] and are not intended to imply that all depicted measurements or clinical outputs are available from a single device [51,52,53,54,55,56].

Representative POC CRP assays have detection limits around 2–2.5 mg/L, whereas some laboratory PCT immunoassays achieve detection limits around 0.02 ng/mL [53,54]. These values illustrate why LOD should not be directly compared across different biomarker classes or interpreted as a measure of diagnostic accuracy. CRP and PCT assays are generally quantitative and require calibration against assay-specific standards, with quality control needed to maintain consistency across runs and provide indirect information about bacterial infection, and should be interpreted within the broader clinical context (Figure 2c). Their principal limitation is not simply analytical sensitivity but biological specificity: CRP and PCT can be altered by noninfectious inflammation, tissue injury, disease severity, and timing. Consequently, their clinical interpretation depends strongly on decision thresholds and context rather than detection alone. POC formats reduce sample-processing and infrastructure requirements, whereas laboratory platforms generally provide broader analytical ranges and more sophisticated calibration and quality-control procedures.

Several technologies are used to detect inflammatory biomarkers at or near the point of care. Conventional laboratory-based PCT and CRP assays commonly rely on antibody-based immunoassay methods, including chemiluminescent, fluorescence, or immunoturbidimetric detection. In these systems, capture antibodies bind the target biomarker, and the resulting optical or luminescent signal is proportional to biomarker concentration. These platforms can provide quantitative results with strong analytical performance, but they often require centralized instruments, trained personnel, and laboratory workflow integration, limiting their usefulness when prescribing decisions must be made within minutes [21,52].

Lateral-flow immunoassays represent a more decentralized approach to biomarker detection. These tests use capillary flow through a membrane containing immobilized antibodies that capture biomarkers such as CRP, PCT, or other inflammatory proteins. Because lateral-flow systems are inexpensive, portable, and easy to operate, they are attractive for urgent-care clinics, outpatient offices, pharmacies, and low-resource settings. However, many lateral-flow assays provide qualitative or semi-quantitative outputs, which may reduce precision compared with laboratory-based platforms. Signal intensity can also vary with sample quality, biomarker concentration, and user interpretation, making confirmatory or algorithmic interpretation important in clinical use [51,55,56].

Emerging biosensing technologies aim to improve biomarker detection by combining rapid turnaround with greater analytical sensitivity. Electrochemical biosensors detect biomarker binding through changes in current, voltage, impedance, or other electrical signals at a functionalized electrode surface. These systems can be miniaturized into low-cost, portable devices and may allow multiplex detection of several inflammatory markers from small blood volumes. Fluorescence-based biosensors use labeled antibodies, nanoparticles, or fluorescent probes to generate optical signals when biomarkers are present. Compared with traditional lateral-flow systems, fluorescence-based detection can improve sensitivity and enable more quantitative measurement, although it may require readers or specialized optical components [55,56]. Representative biomarker detection technologies are summarized in Figure 2.

Microfluidic systems can substantially expand the functionality of biosensors by controlling sample transport, reagent delivery, mixing, reaction time, washing, and waste removal within miniaturized devices. For clinical specimens, this integration is particularly important because the analytical performance of a sensor depends not only on the recognition and transduction mechanisms but also on how the biological sample reaches the sensing interface. Microfluidic architectures can reduce sample and reagent consumption, shorten diffusion distances, and automate sequential assay steps, thereby reducing operator intervention and improving workflow consistency [55,56]. In blood-based bacterial-versus-viral diagnostics, however, sample handling remains challenging because whole blood and serum contain cells, proteins, lipids, and other components that can interfere with target capture and signal generation. Microfluidic separation, filtration, plasma extraction, dilution, and controlled flow can therefore serve as important preprocessing steps before biosensing. Recent microfluidic diagnostic platforms increasingly integrate these functions with pathogen or biomarker detection in compact devices, supporting the development of near-sample-to-answer systems [55,56].

Sensor functionalization and signal transduction are equally important determinants of biosensor performance. Recognition elements, including antibodies, aptamers, nucleic-acid probes, peptides, or other affinity ligands, must be immobilized on the sensing surface while maintaining sufficient target accessibility and minimizing nonspecific interactions. Surface chemistry therefore influences capture efficiency, selectivity, stability, and reproducibility. Following target recognition, the resulting biochemical interaction must be converted into a measurable physical signal. Electrochemical biosensors may measure changes in current, potential, conductance, or impedance, whereas optical systems can quantify changes in fluorescence, absorbance, scattering, or refractive properties [55,56]. For impedance-based sensors in particular, binding events at the electrode interface can alter the local dielectric environment and interfacial electrical properties, allowing label-free detection. These transduction mechanisms offer opportunities for miniaturization and multiplexing, but their practical performance depends strongly on electrode architecture, surface functionalization, signal-to-noise ratio, and stability of the sensing interface.

Biofouling is a major barrier to translating biosensors from controlled buffer experiments to clinical samples. Nonspecific adsorption of abundant serum or plasma proteins and other matrix components onto the sensing surface can block recognition sites, alter interfacial charge or impedance, increase background signal, and reduce assay reproducibility. This challenge is particularly important for label-free electrochemical and impedance-based sensors because nonspecific adsorption can directly generate electrical changes that resemble target-associated signals. Antifouling coatings, optimized blocking strategies, hydrophilic surface chemistries, selective capture layers, sample dilution or pretreatment, and controlled microfluidic transport can help reduce these effects. Consequently, evaluation of biosensor performance in clinically relevant matrices should extend beyond buffered analytical standards and include matrix effects, nonspecific binding, sensor-to-sensor variability, and long-term interface stability. These engineering considerations are essential if rapid biosensors are to generate reliable information within the clinical decision window rather than only demonstrate high analytical performance under laboratory conditions [57,58].

Multiplexing is particularly important for bacterial-versus-viral differentiation because single-analyte detection often provides incomplete information about infectious etiology. Microfluidic devices can spatially separate multiple sensing regions, while multiplex electrochemical or optical architectures can use independently addressable electrodes or detection channels to measure several targets from the same specimen. Such systems can simultaneously evaluate bacterial and viral nucleic acids, pathogen antigens, inflammatory biomarkers, or combinations of pathogen- and host-derived signals. This architecture is potentially more informative for antimicrobial stewardship than a single-target assay because it can provide complementary evidence within one testing workflow. However, increasing the number of analytes also introduces technical challenges, including cross-reactivity, nonspecific binding, competition for reagents, signal overlap, differences in target abundance, channel-to-channel variability, and the need for independent calibration. Spatially separated microfluidic chambers, orthogonal recognition elements, individually addressable electrodes, and algorithmic signal normalization are therefore important strategies for achieving reliable multiplex performance [55,56]. Recent microfluidic biosensor research increasingly focuses on integrating multiplex detection with automated fluid handling and portable readout systems, although comprehensive sample-to-answer multiplex platforms remain an important translational challenge [59,60].

Procalcitonin is one of the most extensively studied biomarkers for bacterial infection. Under normal physiologic conditions, circulating PCT levels remain low; however, during systemic bacterial infection, inflammatory cytokines such as IL-6 and TNF-α can stimulate extra-thyroidal PCT production [61]. Viral infections, in contrast, may be associated with lower PCT responses, providing a biologic basis for bacterial-versus-viral differentiation [18,61]. Many PCT assays rely on automated immunoassay technology, in which antibodies bind circulating PCT and generate measurable signal proportional to biomarker concentration.

Clinically, PCT has been studied extensively as a tool for guiding antibiotic decisions in respiratory infections. Reported diagnostic performance varies depending on infection severity, patient population, and cutoff threshold, and PCT performs best not as an isolated diagnostic classifier but as part of structured antimicrobial stewardship algorithms [18,52]. The ProHOSP randomized trial provides comparatively strong evidence for the clinical utility of PCT when used within an explicit treatment algorithm. PCT-guided management reduced mean antibiotic exposure from 8.7 to 5.7 days compared with standard care, while meeting the prespecified noninferiority criterion for a composite outcome including death, ICU admission, disease-specific complications, and recurrent infection requiring antibiotics. Antibiotic-associated adverse effects were also reduced (19.8% vs. 28.1%). Thus, the stewardship evidence for PCT reflects not simply measurement of a biomarker, but integration of the biomarker result into a predefined antibiotic initiation and discontinuation algorithm [62]. Importantly, the evidence supporting PCT-guided antibiotic reduction derives from structured clinical decision algorithms rather than from analytical performance of the PCT assay alone. Thus, the demonstrated stewardship effect should be attributed to the combination of biomarker measurement, predefined clinical thresholds, and an associated treatment algorithm rather than to PCT detection in isolation [62]. However, important limitations remain. PCT levels may rise in severe trauma, surgery, systemic inflammation, or prolonged shock states, reducing specificity in critically ill populations [18,63]. Additionally, mild localized bacterial infections may not produce sufficiently elevated PCT concentrations for reliable detection.

CRP represents another widely utilized inflammatory biomarker. CRP is synthesized by hepatocytes in response to IL-6-mediated inflammatory signaling and rises rapidly during acute inflammation [64]. POC CRP systems typically use immunoassay-based methods to generate rapid quantitative or semi-quantitative results from blood samples. Evidence that CRP-guided strategies reduce antibiotic prescribing similarly reflects the use of CRP results within clinical decision-making pathways rather than the analytical performance of the CRP assay alone [51]. Therefore, the potential stewardship value of rapid CRP testing should be distinguished from evidence obtained from randomized or prospective prescribing interventions.

However, CRP possesses substantially lower specificity than molecular pathogen-directed diagnostics. Elevated CRP may occur in autoimmune disease, trauma, malignancy, postoperative inflammation, or numerous noninfectious inflammatory conditions [64]. As a result, CRP alone cannot reliably distinguish bacterial from viral disease and instead functions primarily as a probabilistic inflammatory indicator. Nevertheless, because CRP testing is inexpensive, rapid, and operationally simple, it remains attractive for decentralized outpatient and low-resource settings [51,64].

Overall, biomarker-based diagnostics provide a valuable middle ground between highly accurate but resource-intensive molecular testing and rapid but less sensitive antigen-based approaches. By measuring host inflammatory responses, these technologies can generate clinically relevant information within the narrow timeframe in which antibiotic decisions are often made. However, because biomarkers reflect physiologic immune activity rather than direct pathogen presence, their performance may be influenced by factors such as age, immune status, comorbidities, infection severity, and timing of presentation. Consequently, biomarker assays are most effective when integrated into broader clinical decision-making frameworks rather than used as standalone diagnostic tools. Future advances will likely focus on multiplex biosensing platforms that combine lateral-flow, electrochemical, and fluorescence-based detection technologies to improve sensitivity, speed, and clinical utility while maintaining compatibility with POC workflows. Figure 2c highlights the principal tradeoff in biomarker-based diagnostics: single biomarkers provide rapid and accessible measurements but generally have limited etiologic specificity, whereas multi-marker approaches can improve bacterial-versus-viral discrimination by integrating complementary biological signals at the expense of greater analytical and interpretive complexity.

### 3.4. Host-Response Diagnostics

**Figure 3:**
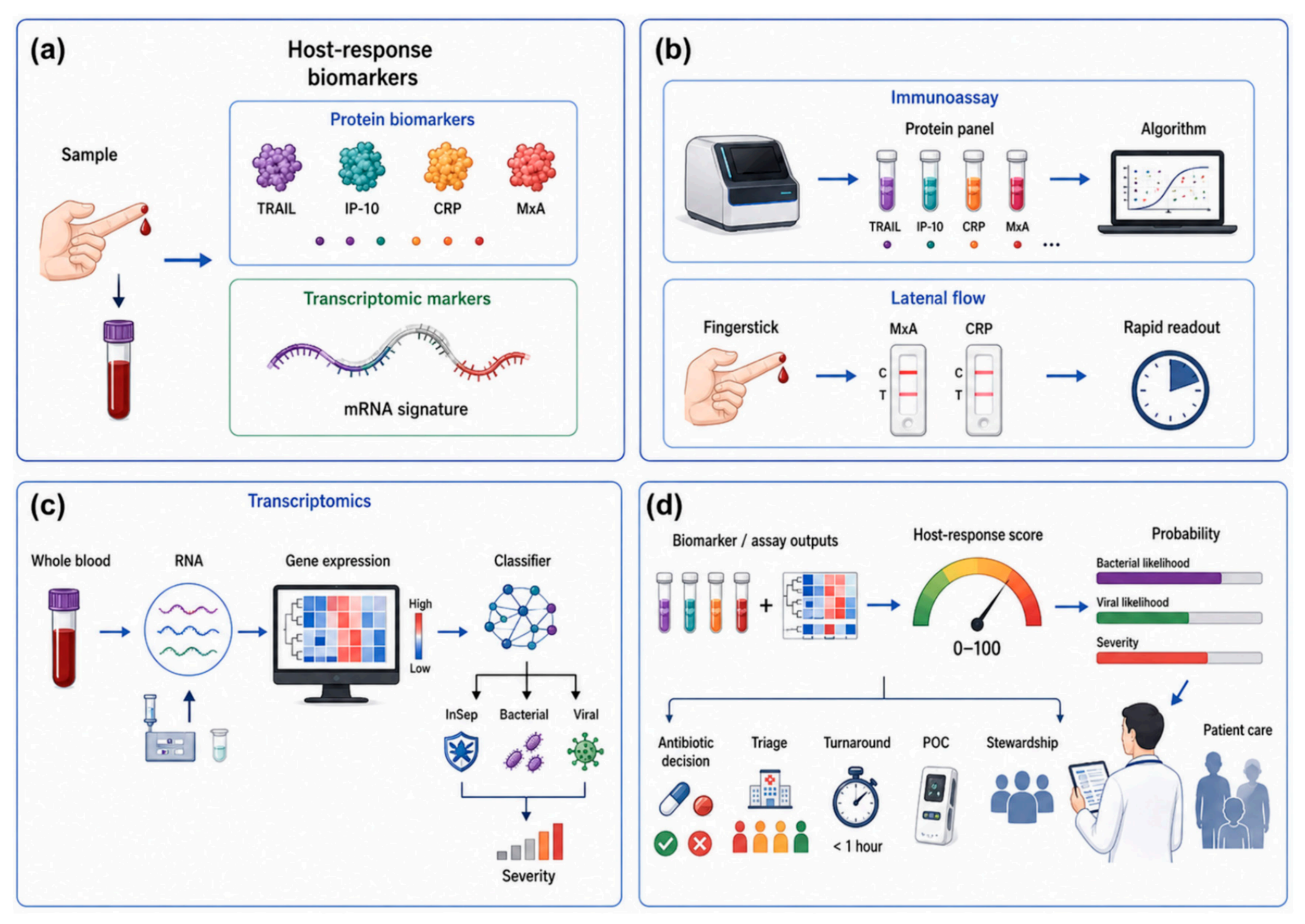
Host-response diagnostic technologies for bacterial-versus-viral infection assessment. All panels are conceptual representations based on the host-response diagnostic literature and do not imply that the depicted measurements, classification outputs, or clinical decisions are generated by a single platform. (**a**) Representative host-response biomarkers measured from blood or fingerstick specimens, including protein markers such as TRAIL, IP-10, CRP, and MxA, together with transcriptomic mRNA signatures. (**b**) Representative protein-based approaches, including multi-marker immunoassays and lateral-flow formats, illustrating different strategies for host-response measurement. (**c**) Conceptual transcriptomic workflow showing whole-blood RNA analysis, gene-expression profiling, and computational classification for infectious-disease assessment. (**d**) Conceptual integration of host-response measurements with clinical information, illustrating how validated host-response classifiers may contribute to bacterial-versus-viral assessment and, separately, how diagnostic information may subsequently inform clinical management and antimicrobial stewardship. These functions represent distinct analytical and clinical steps and should not be interpreted as universal outputs of every host-response platform [65,66,67,68,69,70,71].

Host-response diagnostics address bacterial-versus-viral differentiation from a fundamentally different perspective than pathogen-directed assays. Rather than identifying the infectious organism directly, these approaches measure coordinated changes in the host immune response that may differ according to the underlying infectious etiology. This distinction is particularly relevant to antimicrobial stewardship because host-response signatures may provide information about whether antibacterial therapy is warranted even when a specific pathogen cannot be identified. Importantly, these systems are designed to address the clinically relevant question of whether antibiotic therapy is likely to be beneficial rather than simply identifying the presence of a microorganism [20,65]. Representative host-response diagnostic platforms are summarized in Figure 3.

MeMed BV (MeMed Ltd., Tirat Carmel, Israel) represents a multi-analyte host-response immunoassay in which TRAIL, IP-10, and CRP are measured in parallel and computationally integrated into a bacterial-versus-viral likelihood score [65,66]. The system uses chemiluminescence-based analyte measurement within a disposable cartridge and dedicated analyzer, requiring approximately 100 μL of serum and producing a result in approximately 15 min [67]. The FDA-cleared assay has reported analytical detection limits of 0.067 mg/L for CRP, 4.31 pg/mL for IP-10, and 7.03 pg/mL for TRAIL, although these analytical limits should not be confused with the clinical decision thresholds used by the integrated classifier [67]. The multiplexed architecture is a major advantage because the diagnostic signal arises from coordinated host-response information rather than a single analyte. At the same time, the dedicated analyzer, cartridge-based workflow, calibration/QC requirements, and susceptibility of host-response signals to biological variation increase system complexity compared with simple lateral-flow tests.

The biologic rationale underlying MeMed BV is based on differential immune regulation. TRAIL expression tends to increase during viral infection, while CRP and inflammatory signaling pathways associated with bacterial immune activation are typically elevated during bacterial disease [65,68]. IP-10 contributes additional information related to interferon-mediated antiviral immune responses. By integrating these pathways simultaneously, the assay attempts to overcome the limited specificity associated with single inflammatory biomarkers alone. Clinical studies have reported promising diagnostic performance for MeMed BV in bacterial-versus-viral discrimination [65,66]. However, the clinical interpretation of a low-probability bacterial result is dependent on the validated decision threshold, underlying disease prevalence, patient population, and clinical setting; therefore, negative predictive value should not be interpreted as an intrinsic or universal characteristic of the platform. MeMed BV should consequently be viewed as a probabilistic host-response classification tool rather than a standalone bacterial rule-out test. Its potential contribution to antimicrobial stewardship lies in providing additional evidence that may support withholding or discontinuing antibiotics when consistent with the overall clinical assessment and management pathway, rather than independently determining whether antibiotic treatment is appropriate.

Another rapid host-response platform is FebriDx (Lumos Diagnostics, Sarasota, FL, USA). FebriDx uses two monoclonal-antibody-based lateral-flow assays to detect the host-response proteins MxA and CRP from approximately 5 μL of fingerstick whole blood [70,71,72]. The disposable device integrates the lancet, blood collection, lateral-flow strip, and buffer delivery into a single cartridge, eliminating the need for an external reader. Results are available after approximately 10 min [73,74]. The reported detection thresholds are 40 ng/mL for MxA and 20 mg/L for CRP. This architecture provides excellent manufacturability and decentralization potential, but the qualitative readout sacrifices the quantitative information available from laboratory immunoassays. Interpretation is also influenced by infection timing and host-response heterogeneity, and concurrent bacterial and viral infection cannot necessarily be excluded.

Transcriptomic host-response platforms such as InSep (Inflammatix, Burlingame, CA, USA) differ fundamentally from protein-based POC assays because their biorecognition strategy is based on measurement of host gene-expression signatures rather than individual circulating proteins. The assay uses whole-blood RNA and multiplexed measurement of host-response transcripts followed by computational classification. Recent descriptions of the technology report analysis of 29 host-response genes from approximately 2.5 mL of whole blood using a dedicated cartridge/instrument system with embedded preprocessing and machine-learning algorithms [75]. Because the diagnostic output is derived from relative expression patterns rather than a single analyte concentration, a conventional molecular LOD is not an especially informative summary metric. The principal technical considerations instead include RNA integrity, extraction/amplification efficiency, transcript measurement reproducibility, calibration of the classification model, and robustness to biological and population variability. This architecture enables multiplex host-response classification but requires substantially greater instrumentation and sample-processing complexity than lateral-flow assays.

Unlike pathogen-directed diagnostics, host-response systems are designed to provide probabilistic information about infectious etiology that may be relevant to antibiotic decision-making. Their outputs do not independently determine whether antibiotics are warranted. Instead, interpretation depends on the clinical syndrome, disease severity, likelihood of coinfection, alternative diagnoses, patient characteristics, and the clinical pathway in which the result is used. Consequently, host-response diagnostics are best viewed as clinical decision-support tools rather than autonomous treatment-selection systems. Nevertheless, their outputs remain probabilistic rather than definitive. Immune-response patterns may be altered by coinfection, immunosuppression, chronic inflammatory disease, age-related immune variation, or disease progression stage [20,68]. Consequently, host-response diagnostics are best viewed as advanced clinical decision-support tools rather than standalone diagnostic endpoints. Their major strength lies in their potential to provide rapid, workflow-compatible assessment of infection etiology during the narrow time window in which empiric antibiotic decisions are typically made. However, this temporal and biological advantage should not be equated with demonstrated stewardship benefit. Prospective studies evaluating whether host-response results actually alter antibiotic initiation, discontinuation, duration, or patient outcomes remain necessary to establish their clinical utility [10,21].

Importantly, the potential utility of host-response diagnostics extends beyond respiratory infections to non-respiratory and systemic infectious syndromes, including undifferentiated sepsis, where the infectious source and microbial etiology may remain uncertain during the initial clinical encounter. In these settings, conventional pathogen-directed diagnostics may be limited by delayed turnaround times, prior antimicrobial exposure, low pathogen burden, or difficulty obtaining an appropriate clinical specimen, while nonspecific inflammatory responses may make bacterial and viral infections difficult to distinguish using individual biomarkers alone. Host-response approaches that integrate coordinated immune signatures, transcriptomic profiles, or combinations of inflammatory markers may therefore provide an additional means of estimating infection etiology when direct pathogen detection is unavailable or inconclusive. However, sepsis presents substantial biological heterogeneity, and host-response profiles may be influenced by disease stage, coinfection, immunosuppression, and noninfectious inflammatory conditions. Consequently, although these approaches are promising for resolving diagnostic uncertainty in systemic infections, their ultimate antimicrobial-stewardship value depends on prospective validation and demonstration that the resulting information can safely influence antibiotic initiation, continuation, or de-escalation in real-world clinical workflows.

### 3.5. Hematologic Approaches

**Figure 4:**
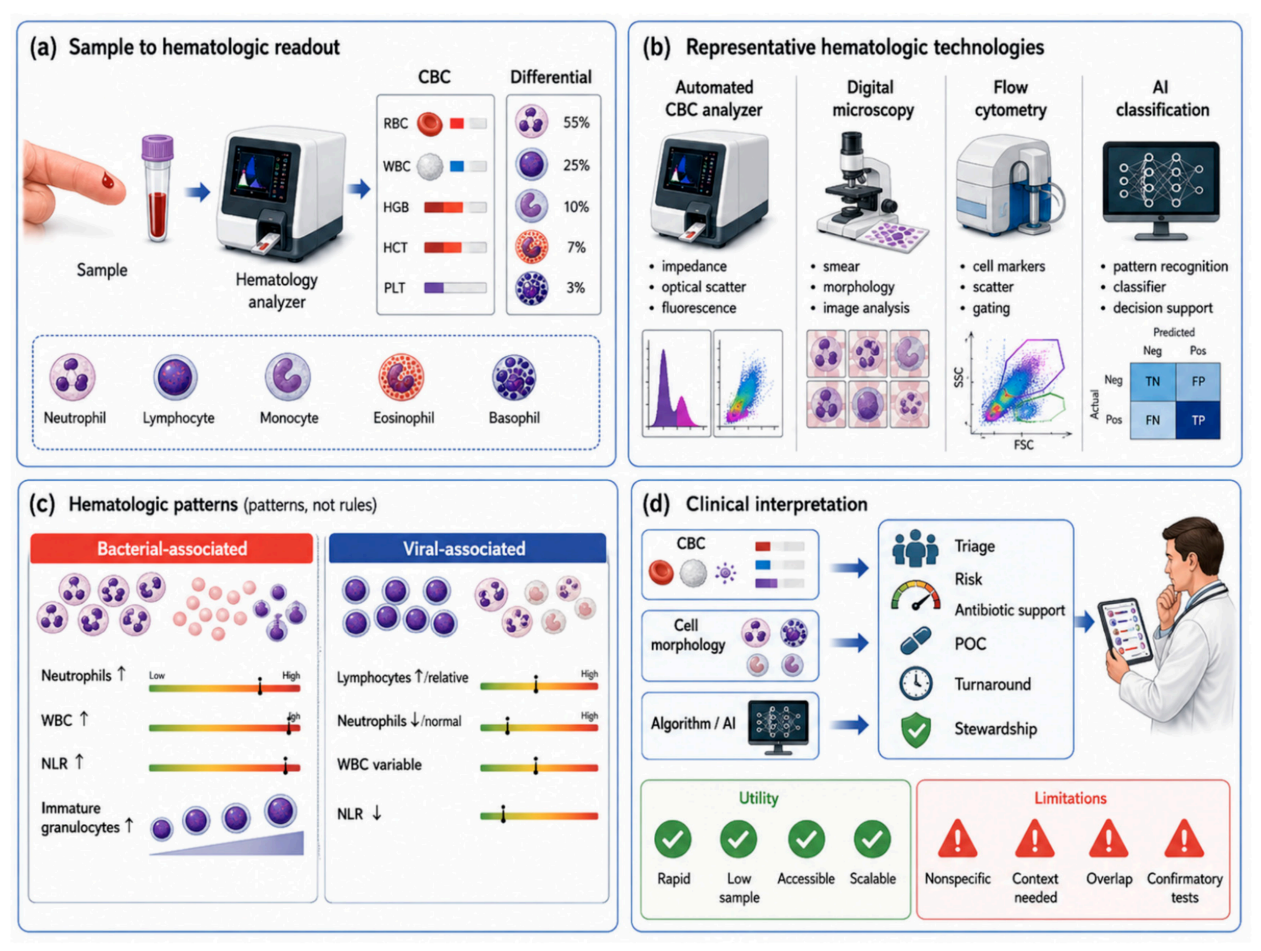
Hematologic approaches for infection assessment and adjunctive clinical decision support. All panels are conceptual representations and do not imply that a single hematology platform performs etiologic classification, severity prediction, or antimicrobial-stewardship decision-making. (**a**) General workflow from blood or fingerstick specimen collection to automated hematologic measurement and reporting of CBC and differential parameters. (**b**) Representative hematologic technologies, including automated hematology analyzers, microscopy-based morphology assessment, flow cytometry, digital imaging, and point-of-care CBC systems. (**c**) Hematologic patterns that may differ between bacterial, viral, and noninfectious inflammatory conditions, including changes in leukocyte counts, neutrophils, lymphocytes, neutrophil-to-lymphocyte ratio, immature granulocytes, platelets, and monocytes. These patterns are nonspecific and require clinical contextualization. (**d**) Conceptual representation of how hematologic measurements may serve as adjunctive information within broader clinical assessment and multimodal diagnostic workflows. The figure does not imply that hematologic measurements alone establish bacterial-versus-viral etiology or directly determine antibiotic treatment [76,77,78,79,80,81,82,83,84,85].

Hematologic diagnostics provide an indirect approach to bacterial-versus-viral differentiation by analyzing changes in circulating immune-cell populations rather than directly detecting pathogens or measuring pathogen-specific biomarkers. These approaches primarily rely on complete blood count (CBC) parameters and white blood cell (WBC) differentials, which reflect dynamic host responses during infection. Bacterial infections frequently produce neutrophil-dominant responses, whereas some viral infections are associated with relative lymphocytic responses or other changes in leukocyte distributions [76,77]. CBC-derived measurements can therefore provide rapid contextual information about the host response during an infectious presentation, although substantial overlap exists between bacterial, viral, and noninfectious inflammatory conditions. Recent advances in POC hematology have enabled near-patient measurement of these parameters using small blood volumes and short turnaround times. Representative hematologic diagnostic approaches are summarized in Figure 4.

Sight OLO (Sight Diagnostics Ltd., Tel Aviv, Israel) provides automated CBC and differential measurements using optical/cell-analysis methods rather than molecular or immunochemical target recognition. The platform requires a small whole-blood specimen and a dedicated compact analyzer, with analytical measurement ranges established for WBC, hemoglobin, and platelet parameters; for example, the reported WBC linearity range extends from approximately 0.18 to 100.13 × 10^3^/µL [78]. Because the system measures cellular populations rather than a specific molecular analyte, conventional biosensor LOD is not directly applicable. Quality control and calibration are instead centered on hematology analyzer performance, cell-count accuracy, and control materials. The platform also has documented interference evaluations for substances including bilirubin, hemoglobin, chyle, and intralipids [78]. These characteristics support rapid decentralized CBC testing, but the measured hematologic patterns remain nonspecific and should not be interpreted as direct bacterial-versus-viral detection. These capabilities demonstrate the analytical and operational feasibility of obtaining detailed hematologic measurements near the point of care, but they do not by themselves establish bacterial-versus-viral etiology or demonstrate a reduction in antibiotic prescribing.

The biologic rationale for using hematologic patterns in infectious disease assessment is based on differences in leukocyte responses between infection types. Bacterial infections frequently produce neutrophilia and elevated absolute neutrophil counts, whereas some viral infections may produce relative lymphocytosis or reduced neutrophil predominance [76,77]. However, these patterns are neither sufficiently specific nor sufficiently consistent to independently distinguish bacterial from viral infection. Their interpretation is influenced by infection stage, age, corticosteroid exposure, underlying inflammatory or malignant disease, immunosuppression, physiologic stress, and other patient-specific factors. Consequently, CBC-derived patterns should be interpreted as supportive evidence within a broader diagnostic assessment, rather than as definitive etiologic classification.

Another emerging POC hematology platform, HemoScreen (PixCell Medical Technologies, Yokneam Illit, Israel), uses disposable cartridges and imaging-based analysis to perform automated CBC and five-part differential testing from small blood samples [79,80]. The platform is designed to generate rapid hematologic results and has demonstrated substantial agreement with central laboratory hematology systems across major CBC parameters [79,80]. Its compact format and simplified operation may facilitate rapid CBC assessment in emergency departments, outpatient clinics, and resource-limited environments. However, these analytical and workflow characteristics should not be interpreted as evidence that HemoScreen independently improves antimicrobial prescribing or clinical outcomes. Importantly, these platforms provide rapid hematologic measurements rather than direct bacterial-versus-viral classification; their potential value is therefore primarily as an adjunctive source of information within a broader clinical and diagnostic assessment. To date, rapid availability of CBC-derived measurements should therefore be considered a workflow and information-access advantage rather than a demonstrated antimicrobial-stewardship intervention.

Additional hematologic POC technologies include systems such as HemoCue WBC DIFF (HemoCue AB, Ängelholm, Sweden), which uses microcuvette-based blood sampling and optical analysis to generate rapid WBC and differential measurements [81]. These platforms prioritize operational simplicity and rapid turnaround while providing less comprehensive information than large laboratory hematology analyzers. Because CBC testing is already routinely incorporated into clinical evaluation, rapid availability of differential counts may facilitate integration with existing clinical workflows. This represents a workflow advantage rather than a demonstrated antimicrobial-stewardship effect, and the extent to which rapid CBC information changes antibiotic decisions depends on how the results are incorporated into clinical assessment and treatment pathways.

Despite their accessibility and rapid turnaround, hematologic diagnostics remain inherently nonspecific. Leukocyte patterns are influenced not only by infection type but also by age, corticosteroid exposure, autoimmune disease, physiologic stress, trauma, malignancy, immunosuppression, and disease stage [76,77]. Significant overlap between bacterial and viral hematologic responses further limits their ability to provide definitive etiologic classification. In addition, CBC-derived patterns provide physiologic inference rather than direct pathogen confirmation (Figure 4c). Consequently, hematologic systems should be viewed primarily as supportive and triage-oriented measurements that may complement clinical assessment, targeted pathogen testing, biomarkers, or host-response diagnostics.

Importantly, the evidence supporting hematologic POC systems differs from that supporting biomarkers such as CRP. Randomized trials and systematic reviews have demonstrated that CRP POCT, when used as an adjunct to clinical decision-making and prescribing guidance, can reduce antibiotic use in selected primary-care populations without compromising recovery [82,83]. In contrast, the studies supporting the hematologic platforms discussed above primarily evaluate analytical agreement, measurement performance, and operational feasibility [79,80,81,84,85]. Comparable interventional evidence demonstrating that CBC-differential POCT alone reduces unnecessary antibiotic prescribing or improves infectious-disease outcomes is currently limited. Therefore, the potential antimicrobial-stewardship role of rapid hematologic testing should be considered conditional on integration with clinical assessment and complementary diagnostic information, rather than assumed from its rapid availability.

However, hematologic diagnostics possess several important advantages within antimicrobial stewardship workflows. They are rapid, minimally invasive, and highly compatible with existing outpatient and emergency-care infrastructure. Unlike many molecular assays requiring specialized consumables or extended processing times, POC hematology systems can deliver clinically relevant information within minutes using small-volume blood samples. As a result, hematologic diagnostics may be particularly valuable when integrated into broader multimodal diagnostic frameworks designed to reduce unnecessary empiric antibiotic prescribing during early clinical decision-making [10,21].

### 3.6. AI-Based Tools

Artificial intelligence (AI) and machine-learning systems are increasingly being integrated into infectious disease management as clinical decision-support tools. Unlike molecular, antigen, or host-response diagnostics, AI-based systems generally do not generate new biologic measurements themselves. Instead, they analyze combinations of existing clinical data, including laboratory values, vital signs, imaging findings, electronic health record (EHR) variables, and demographic characteristics, to identify patterns associated with infection severity, sepsis risk, or likelihood of bacterial disease. Most platforms rely on supervised machine-learning models trained on large retrospective clinical datasets to generate probabilistic predictions that may assist clinician decision-making [86,87]. Within the context of bacterial-versus-viral differentiation and antimicrobial stewardship, the relevance of AI depends on whether the system merely predicts infection or sepsis risk, or whether it integrates diagnostic information to estimate infectious etiology and support antibiotic decision-making.

One of the most widely studied AI-assisted sepsis detection systems is TREWS (Targeted Real-time Early Warning System)(Bayesian Health, New York, NY, USA), developed using machine-learning algorithms trained on EHR-derived physiologic and laboratory variables [88]. TREWS continuously analyzes incoming patient data, including temperature, heart rate, respiratory rate, blood pressure, WBC count, lactate concentration, and prior clinical history, to identify patterns associated with evolving sepsis [88,89]. Rather than functioning as a simple threshold-based alert system, the platform applies predictive modeling to estimate sepsis risk dynamically as new data become available. Clinical evaluations have reported that TREWS-assisted workflows were associated with earlier sepsis recognition and faster antibiotic administration in hospitalized patients [89]. However, because TREWS primarily predicts risk of severe bacterial infection rather than distinguishing bacterial from viral disease directly, its role in antimicrobial stewardship remains indirect.

The Epic Sepsis Model (ESM) (Epic Systems Corporation, Verona, WI, USA) represents another widely implemented machine-learning-based clinical prediction system. ESM integrates EHR variables including vital signs, laboratory trends, medication history, and nursing documentation to generate automated sepsis risk scores within hospital workflows [90]. Unlike pathogen-directed diagnostics, these systems function through multivariable statistical pattern recognition rather than biologic target detection. However, independent validation studies have demonstrated substantial variability in ESM performance across healthcare systems, with reported concerns regarding sensitivity, false-positive burden, and generalizability [90]. One major limitation of AI-assisted clinical prediction models is that algorithm performance may decline when applied outside the populations or hospital systems on which training datasets were originally developed. These systems should be distinguished from etiologic classifiers because they primarily estimate sepsis risk or clinical deterioration rather than determine whether an infection is bacterial or viral. Their potential to facilitate earlier recognition and treatment therefore represents a different clinical objective from antimicrobial stewardship aimed at avoiding unnecessary antibiotic exposure.

AI approaches have also been explored specifically for bacterial-versus-viral differentiation. Machine-learning models incorporating CBC parameters, inflammatory biomarkers, radiographic findings, clinical variables, and host-response transcriptomic data have demonstrated promising diagnostic performance in research settings [91,92]. For example, transcriptomic machine-learning classifiers trained on host gene-expression signatures have been investigated for distinguishing bacterial from viral infections, while other experimental systems combine CRP, procalcitonin, neutrophil-to-lymphocyte ratio, and clinical metadata into ensemble prediction models designed to support antibiotic decision-making [18,76]. These approaches are particularly relevant to the present review because they move beyond sepsis-risk prediction toward etiologic classification, potentially allowing AI systems to synthesize multiple weak or complementary biological signals into a single estimate of bacterial-versus-viral likelihood. Nevertheless, many such systems remain investigational, and evidence demonstrating that their predictions directly reduce unnecessary antibiotic prescribing or improve antimicrobial de-escalation remains more limited than evidence of diagnostic performance alone.

A particularly promising direction is the integration of AI with biosensor and multimodal diagnostic platforms. Rapid biosensors can generate continuous or high-dimensional signals that may contain diagnostically relevant information beyond a single endpoint measurement. In this context, device-level multiplexing and high-dimensional electrochemical or optical measurements may provide richer input features than binary endpoint assays, although the resulting models must be trained and validated against clinically meaningful outcomes rather than analytical sensor signals alone. Machine-learning algorithms can potentially extract features from impedance spectra, electrochemical responses, multiplexed biomarker profiles, or other sensor outputs and combine them with complementary clinical measurements to generate a more comprehensive assessment of infection. Such biosensor-integrated AI approaches could ultimately support automated interpretation at the point of care and provide clinicians with an actionable bacterial-versus-viral assessment within the relevant treatment-decision window.

**Figure 5:**
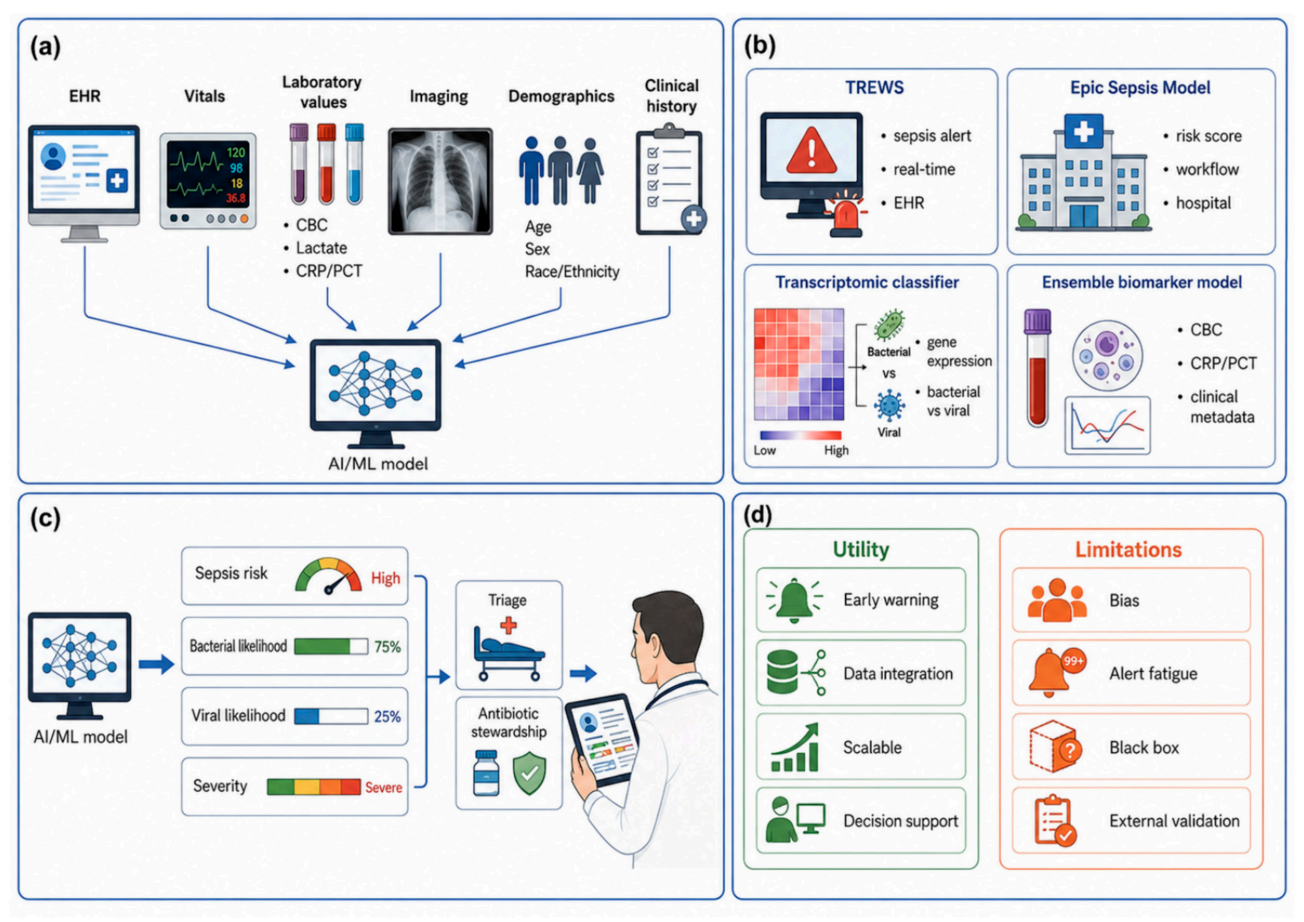
AI-based clinical decision-support tools for infectious disease assessment. (**a**) Multimodal clinical inputs used by AI and machine-learning models include EHR data, vital signs, laboratory values, imaging findings, demographic variables, and clinical history. (**b**) Representative AI-based approaches, including real-time sepsis alert systems such as TREWS, EHR-integrated risk models such as the Epic Sepsis Model, transcriptomic classifiers, and ensemble biomarker models combining CBC, inflammatory markers, and clinical metadata. (**c**) Examples of outputs generated by different AI approaches, including sepsis-risk prediction, bacterial-versus-viral likelihood estimation, or infection-severity prediction. These outputs are conceptual and are not necessarily generated simultaneously by a single platform. (**d**) Key clinical utilities and limitations of AI-based systems, including early warning, data integration, scalability, and decision support, as well as concerns related to bias, alert fatigue, black-box interpretation, and the need for external validation.

Despite their potential, AI-based diagnostic systems face several important limitations. Machine-learning algorithms are highly dependent on training-data quality, population diversity, and external validation. Biases within training datasets may reduce diagnostic reliability across different demographic groups or healthcare environments [86,87]. In addition, continuous alert generation may contribute to alert fatigue, reducing clinician responsiveness and limiting practical implementation effectiveness [90]. Many AI systems also function as “black-box” models, making clinical interpretation difficult when prediction rationale is not transparent. Importantly, high predictive performance does not by itself establish clinical utility or antimicrobial-stewardship benefit. For this reason, AI-based bacterial-versus-viral classifiers should ultimately be evaluated in prospective clinical studies using antibiotic initiation, discontinuation, treatment duration, patient safety, hospitalization, and mortality as clinical endpoints rather than diagnostic accuracy alone. For bacterial-versus-viral classification systems, prospective evidence is still needed to determine whether model outputs change antibiotic initiation, continuation, or de-escalation without increasing the risk of undertreating bacterial infection. AI tools therefore should currently be viewed primarily as decision-support technologies that augment clinical and diagnostic information rather than as replacements for microbiological or host-response testing. Representative AI-based clinical decision-support approaches for infectious disease assessment are summarized in Figure 5.

## 4. Comparative Analysis

Comparison of current rapid diagnostic technologies reveals a persistent tradeoff between analytical performance, operational feasibility, and clinical actionability. Although molecular platforms such as GeneXpert [27,28], BioFire RP2.1 [35,36], and other PCR-based systems provide high analytical and clinical diagnostic performance for their predefined targets, these measures primarily establish pathogen detection and do not by themselves establish bacterial-versus-viral classification or reduced antibiotic use. Clinical sensitivity and specificity should be interpreted relative to the specific reference standard and target population, while the potential stewardship value depends on whether results are available before or during the antibiotic decision. In many outpatient and emergency settings, treatment decisions may occur before molecular results become available, limiting the ability of even highly sensitive assays to reduce empiric antibiotic prescribing [15,16,21,93]. Multiplex panels can provide broader etiologic information than single-pathogen assays, but evidence that molecular testing alone changes antibiotic prescribing or patient outcomes remains dependent on clinical workflow and implementation strategy.

In contrast, rapid antigen tests and POC biomarker assays prioritize accessibility and workflow compatibility over maximal analytical precision. Technologies such as Abbott BinaxNOW [37,39], BD Veritor [17,38], and Quidel QuickVue Strep A [43] provide results within clinically relevant decision windows and require minimal infrastructure, making them operationally attractive in decentralized settings. However, reduced sensitivity, particularly during early infection or low pathogen burden, limits their reliability as standalone rule-out tools [17,37]. Similarly, biomarkers such as CRP [51,64] and procalcitonin [18,52] provide indirect inflammatory information that must be interpreted within a broader clinical context.

For rapid antigen tests, analytical and clinical performance should similarly be distinguished from clinical impact. A positive result may provide actionable evidence for a predefined pathogen, whereas a negative result does not necessarily exclude infection when sensitivity is limited. Therefore, agreement with molecular testing or culture establishes diagnostic performance but does not demonstrate that the test reduces unnecessary antibiotic prescribing. Demonstrated stewardship benefit depends on how results are incorporated into clinical decision pathways, including whether negative results trigger confirmatory testing or alter antibiotic initiation.

Host-response diagnostics occupy an intermediate position between pathogen-directed testing and generalized inflammatory biomarkers. Platforms such as MeMed BV [65,66], FebriDx [70,71], and emerging transcriptomic systems, including InSep [91,92], more directly address the clinically relevant question of bacterial-versus-viral etiology. These technologies may have potential stewardship advantages because they evaluate coordinated immune-response patterns rather than detecting isolated microbial targets; however, whether this translates into reduced antibiotic exposure depends on prospective implementation and the clinical decision pathway. However, their outputs remain probabilistic, and broader implementation is currently limited by cost, platform complexity, and the need for large-scale prospective validation across diverse patient populations [20,68].

Although MeMed BV directly addresses bacterial-versus-viral likelihood through a host-response signature, its diagnostic classification performance should be distinguished from demonstrated changes in antibiotic prescribing or patient outcomes. The platform therefore represents a promising etiologic decision-support approach, while the magnitude of its antimicrobial-stewardship benefit depends on prospective implementation and the clinical decision pathway in which the result is used. FebriDx similarly provides host-response information relevant to bacterial-versus-viral differentiation, but diagnostic accuracy alone does not establish a reduction in antibiotic use. Its potential stewardship value depends on whether clinicians receive and act upon the result before antibiotic prescribing and whether the test is incorporated into an appropriate clinical decision pathway.

POC hematologic systems introduce an additional workflow-oriented approach by leveraging routinely available CBC-derived immune-cell patterns. Technologies such as Sight OLO [84,85] and PixCell HemoScreen [79,80] provide rapid hematologic assessment with limited infrastructure requirements, making them particularly compatible with outpatient and urgent-care environments. Although these systems lack the specificity of molecular or host-transcriptomic platforms, their speed and operational simplicity may provide meaningful clinical utility during early triage and antibiotic decision-making. For hematologic POC systems, analytical agreement with laboratory CBC measurements establishes measurement performance but does not establish bacterial-versus-viral discrimination or antibiotic stewardship benefit. Their potential clinical contribution is therefore adjunctive, and evidence for prescribing or outcome effects should not be inferred from hematologic measurement accuracy alone.

AI-based systems represent a distinct category because their value depends on how effectively they integrate heterogeneous information and translate predictions into clinical decisions. Sepsis early-warning systems, such as TREWS [88,89] and the Epic Sepsis Model [90], primarily address recognition and risk stratification and therefore have an indirect relationship with bacterial-versus-viral differentiation. In contrast, multimodal machine-learning models incorporating biomarkers, hematologic parameters, clinical variables, imaging, or host-response signatures can directly address etiologic classification. A further distinction should be made between diagnostic performance and demonstrated stewardship impact. Although several AI approaches show promising bacterial-versus-viral classification performance, comparatively fewer studies have prospectively demonstrated that these predictions reduce inappropriate antibiotic initiation, improve antibiotic selection, or facilitate safe de-escalation. Similarly, sepsis early-warning systems primarily evaluate recognition or risk stratification rather than microbial etiology, and their effects on antibiotic use should therefore be evaluated as workflow and implementation outcomes rather than inferred from predictive discrimination alone. Thus, AI-based diagnostics should currently be considered a rapidly developing decision-support category rather than a mature solution for antimicrobial stewardship [86,90].

Collectively, these findings highlight that diagnostic evidence should be interpreted at several distinct levels. Analytical performance describes how reliably a platform detects or measures its target under defined assay conditions, whereas clinical sensitivity and specificity describe performance in the intended patient population relative to a specified reference standard. Neither metric alone establishes bacterial-versus-viral etiologic discrimination, particularly for pathogen-specific tests or nonspecific host-response biomarkers. Clinical utility represents a further level of evidence and depends on whether the result becomes available within the relevant decision window and leads to a change in antibiotic initiation, continuation, discontinuation, or duration. The strongest evidence for antimicrobial stewardship therefore comes from prospective clinical or implementation studies demonstrating changes in prescribing or patient outcomes, rather than from analytical validation or agreement with a laboratory comparator alone. Accordingly, throughout this review, we distinguish analytical and clinical diagnostic performance from demonstrated stewardship impact. When prospective intervention or implementation evidence is unavailable, the potential clinical or stewardship relevance of a technology is described as such rather than presented as an established benefit.

To facilitate clinically meaningful comparison, technologies in Table 1 are grouped according to their primary diagnostic task rather than treated as interchangeable diagnostic modalities. Pathogen-directed assays identify predefined microbial targets; host-response and biomarker assays provide indirect or probabilistic information regarding infectious etiology; and hematologic and computational systems provide adjunctive measurements or risk prediction. Diagnostic performance is reported using the reference standard employed in the corresponding study, and manufacturer/regulatory performance data are distinguished from independent prospective evidence. Where quantitative clinical performance or stewardship outcomes are not available or are not directly comparable, values are reported as not available rather than inferred from heterogeneous studies.

**Table 1:** Quantitative and clinically contextualized characterization of representative rapid diagnostic technologies relevant to bacterial-versus-viral assessment and antimicrobial stewardship. Technologies are grouped according to their primary clinical task: pathogen-directed detection, etiologic/host-response classification, and adjunctive or severity-oriented assessment. Each row represents a defined platform or assay and is linked to a traceable evidence source. Diagnostic-performance estimates are reported within the population, specimen, reference standard, and study design of the cited evaluation and are not intended as direct head-to-head comparisons across technologies. Sensitivity/specificity or PPA/NPA values are accompanied by 95% confidence intervals where reported. For multiplex platforms, performance may vary substantially by analyte, and overall panel-level estimates should not be interpreted as uniform across targets. NR, not reported; N/A, not applicable. Manufacturer and regulatory information is distinguished from independent clinical evidence.

**Pathogen-Directed Tests**	**Technology**	**Intended Use/Population**	**Specimen**	**Reference Standard**	**Sensitivity/PPA**	**Specificity/NPA**	**LOD**	**Sample-to-Result**	**Regulatory Status**	**Evidence Source**	**Stewardship Evidence**
	Cepheid Xpert Xpress Strep A [27,28]	Symptomatic patients with suspected GAS pharyngitis	Throat swab	Bacterial culture	99.4% (95% CI 96.5–99.9)	94.1% (95% CI 91.6–95.9)	~9–18 CFU/mL	18–24 min	FDA-cleared; CLIA-waived	Manufacturer/ clinical evaluation	Direct stewardship evidence is limited; rapid confirmation may support appropriate treatment decisions
	Roche cobas Strep A/cobas Liat [30]	Patients with suspected GAS pharyngitis	Throat swab	Culture	97.7% (95% CI 93.4–99.2)	93.3% (95% CI 89.9–95.6)	5–20 CFU/mL	≤15 min	FDA-cleared/ CLIA-waived	Independent prospective study + manufacturer	Direct prescribing-impact evidence limited; potential to reduce unnecessary empiric treatment by rapid confirmation
	Abbott ID NOW Strep A 2 [37,39]	Patients with suspected GAS pharyngitis	Throat swab	Culture	98.5% (95% CI 95.6–99.5)	93.4% (95% CI 91.4–94.9)	~312–1250 CFU/swab in analytical evaluation	2–6 min	FDA-cleared/ CLIA-waived	Manufacturer clinical data + independent evaluation	Direct stewardship evidence limited
	BioFire FilmArray RP2.1 [35,36]	Patients with suspected acute respiratory infection	Nasopharyngeal swab	Composite/reference molecular methods	Overall panel: 97.1%; analyte-specific performance varies	Overall panel: 99.3%; analyte-specific performance varies	Target-dependent	~45 min	FDA De Novo authorization; US	Multicenter clinical evaluation + manufacturer IFU	Potential stewardship benefit; evidence is implementation-dependent, and pathogen detection does not by itself establish bacterial causation
	Abbott BinaxNOW Influenza A & B [37,39]	Patients with suspected influenza	Nasal/nasopharyngeal specimen	RT-PCR/reference molecular method	Study-dependent; representative prospective study: 29.6% (95% CI 13.8–50.2)	100% (95% CI 98.0–100)	Manufacturer/assay-dependent	~15 min	FDA-cleared	Independent prospective evaluation + FDA documentation	Direct antibiotic-reduction evidence is limited; viral confirmation may support antibiotic avoidance in appropriate clinical pathways
	BD Veritor System for Rapid Detection of Flu A + B [17,38]	Patients with suspected influenza	Nasal/nasopharyngeal specimen	RT-PCR	78% overall in a representative primary-care study	93% overall	Manufacturer/assay-dependent	~10–15 min	FDA-cleared; CLIA-waived versions available	Independent clinical evaluation + FDA documentation	Direct stewardship evidence limited
**Etiologic/** **host-response classifiers**	MeMed BV [65,66,69]	Patients with suspected acute infection, evaluated in ED/acute-care and sepsis populations	Whole blood/serum	Expert clinical reference classification	Example sepsis cohort: 98.8% (95% CI 93.6–100)	89.7% (95% CI 85.3–93.2)	N/A; biomarker-specific	~15 min	FDA-cleared	Independent prospective studies + manufacturer documentation	Clinical-utility evidence exists, but the stewardship effect depends on implementation and the clinical pathway
	FebriDx [70,71]	Acute respiratory illness; intended to aid bacterial vs non-bacterial differentiation	Fingerstick whole blood	Composite Clinical Reference Algorithm	93.2% (95% CI 84.9–97.0)	88.4% (95% CI 85.0–91.0)	MxA/CRP assay-specific	~10 min	FDA 510(k) cleared; CLIA waived in US	Multicenter prospective study + FDA documentation	Direct diagnostic evidence; antibiotic-prescribing impact should be reported separately from diagnostic performance
	InSep/multi-mRNA host-response classifier [91,92,94]	Suspected acute infection/sepsis; intended for bacterial/viral differentiation and severity assessment	Whole blood	Clinical adjudication/reference classification	Study-specific/not sufficiently standardized for a single table value	Study-specific/not sufficiently standardized	N/A	Platform-dependent/developmental	Emerging; regulatory status should be stated as development-stage unless a current authorization is documented	Clinical-development literature	Potential clinical utility; direct stewardship outcomes remain limited
**Adjunctive/** **hematologic/** **severity-oriented tools**	**Technology**	**Intended Use/Population**	**Specimen**	**Reference Standard**	**Clinical Performance**		**LOD**	**Sample-to-Result**	**Regulatory Status**	**Evidence Source**	**Stewardship/ ** **clinical Utility**
	Sight OLO [84,85]	CBC/5-part differential in clinical laboratory/POC	Capillary or venous whole blood	Laboratory hematology analyzer/ microscopy	Strong agreement with reference CBC analyzer; r >0.9 for most CBC parameters in representative evaluation		N/A	<10 min	FDA 510(k) K211840; CE-marked	Independent analytical evaluation + FDA documentation	No direct evidence establishing bacterial-vs-viral classification or antibiotic reduction
	PixCell HemoScreen [79,80]	CBC/5-part differential in POC settings	Capillary/venous whole blood	Central laboratory hematology analyzer	Strong analytical agreement; r >0.90–0.95 for evaluated parameters in representative studies		N/A	~5 min	FDA 510(k) cleared; moderate-complexity POC	Independent analytical evaluations + FDA documentation	No direct evidence establishing bacterial-vs-viral classification or antibiotic reduction
	TREWS [88,89]	Early warning/risk prediction for sepsis	EHR + clinical data	Sepsis diagnosis/clinical recognition	Performance is model-, threshold-, and population-dependent; not an etiologic classifier		N/A	Continuous/real-time	Software/EHR decision-support system; not an IVD	Independent clinical evaluations	May support earlier recognition and antibiotic administration; not demonstrated as an antibiotic-stewardship classifier
	Epic Sepsis Model (ESM) [90]	Prediction of sepsis risk in hospitalized patients	EHR-derived clinical data	Sepsis diagnosis	External validation: AUROC 0.63 (95% CI 0.62–0.64) in one large retrospective cohort; newer ESM v2 multicenter validation reports AUROC 0.82–0.92 across four systems		N/A	Continuous/real-time	Clinical decision-support software; status depends on deployment/regulatory framework	Independent external validation studies	Evidence concerns sepsis recognition/workflow rather than bacterial-vs-viral discrimination; one before–after study associated implementation with lower mortality and faster antibiotic administration

To facilitate interpretation, Table 1 separates technologies according to the clinical question they address rather than treating all rapid diagnostic platforms as competing solutions. Pathogen-directed assays identify predefined microorganisms, host-response classifiers estimate the probability of bacterial versus viral infection, and adjunctive hematologic or computational tools provide complementary information about inflammation or disease severity. The quantitative values are therefore intended to characterize the evidence available for each defined platform rather than establish a universal performance ranking.

Evidence for clinical utility varies substantially across diagnostic categories. The strongest evidence currently comes from biomarker-guided treatment strategies, particularly procalcitonin, where randomized trials have evaluated antibiotic exposure alongside clinical safety outcomes. In the ProHOSP trial, PCT-guided management reduced mean antibiotic exposure from 8.7 to 5.7 days while maintaining similar rates of death, ICU admission, disease-specific complications, and recurrent infection; antibiotic-associated adverse effects were also lower in the PCT group [62]. In critically ill patients, a separate randomized trial similarly found that PCT guidance reduced antibiotic consumption and treatment duration without evidence of harm and reported lower 28-day mortality [95]. These findings support PCT as an example of a diagnostic biomarker whose stewardship value has been evaluated through an explicit treatment algorithm rather than inferred solely from diagnostic performance. Importantly, the stewardship evidence associated with a diagnostic technology should not be extrapolated from its diagnostic performance alone; demonstrated effects on antibiotic prescribing require evaluation within a defined clinical decision pathway, whereas diagnostic classifiers without intervention evidence should be regarded as potentially informative rather than proven stewardship interventions.

In contrast, evidence for many pathogen-directed rapid tests is less consistent. A recent systematic review and meta-analysis of 25 randomized trials involving 12,638 patients found that molecular point-of-care testing for acute respiratory tract infections probably had little to no overall effect on antibiotic use or treatment duration, although it increased appropriate antibiotic prescribing. The intervention also did not significantly affect 30-day mortality or ICU admission [46]. These findings illustrate that rapid availability of microbiological information does not necessarily translate into reduced antibiotic exposure. The effect of testing depends on how results are interpreted, whether they are available before treatment decisions, and whether they are integrated into explicit prescribing or stewardship pathways.

Evidence for other emerging platforms remains more limited. Host-response, hematologic, biosensor, and AI-based approaches increasingly demonstrate promising bacterial-versus-viral classification performance, but prospective evidence showing reductions in antibiotic initiation, duration, discontinuation, reconsultation, hospitalization, adverse events, or mortality remains comparatively sparse. Accordingly, throughout this review, diagnostic performance is distinguished from demonstrated clinical utility, and technologies without direct intervention evidence are described as having potential or proposed stewardship value rather than established benefit.

Current diagnostic technologies exhibit important trade-offs across turnaround time, analytical performance, cost, and ability to inform real-time clinical decision-making. Molecular platforms provide high diagnostic accuracy but are often limited by infrastructure requirements and turnaround time. In contrast, POC approaches such as antigen tests and biomarkers offer faster results but reduced diagnostic precision. Host-response and hematologic methods aim to bridge this gap by providing clinically actionable information within the decision window, though they may remain probabilistic and context-dependent.

From a biosensor and device-development perspective, the technologies also differ substantially in sample preparation, recognition strategy, transduction, multiplexing, and integration requirements. Molecular platforms generally provide the highest analytical sensitivity and can integrate automated extraction, amplification, and detection, but require dedicated instruments and consumables. Antigen-based lateral-flow assays minimize sample preparation and reader requirements and are highly manufacturable, although their lower analytical sensitivity can limit rule-out performance. Biomarker and host-response assays occupy an intermediate position: protein-based systems can provide rapid quantitative or qualitative measurements, whereas transcriptomic platforms offer highly multiplexed biological information but require substantially more complex sample processing and computational infrastructure. Hematologic analyzers provide rapid multi-parameter cellular measurements with relatively small sample volumes but do not have a conventional molecular LOD and should be regarded as adjunctive rather than etiologic diagnostics. Across all categories, matrix effects, biological variability, calibration, internal quality control, and reader requirements can influence real-world performance as much as the nominal analytical sensitivity. Thus, device readiness should be evaluated not only by LOD or accuracy but also by sample-to-answer integration, manufacturability, workflow compatibility, and the maturity of clinical validation.

For antimicrobial stewardship, the most relevant technical configuration is therefore not necessarily the platform with the lowest LOD, but the one that combines sufficient analytical performance with rapid turnaround, appropriate multiplexing or etiologic information, minimal sample handling, and a result that can be incorporated into the clinical decision window.

Emerging label-free optical and biophysical approaches provide complementary strategies for infectious-disease characterization, although their current clinical roles differ substantially from those of established pathogen-directed and host-response assays. Optical coherence tomography (OCT) and dynamic OCT can characterize tissue structure and temporal optical changes without exogenous labeling and have demonstrated infection-associated tissue patterns in application-specific settings such as infectious keratitis; however, these approaches primarily provide host/tissue phenotyping rather than direct pathogen identification, and clinical validation for generalized bacterial-versus-viral classification remains limited [96,97]. Digital holographic microscopy (DHM), digital holography, quantitative phase imaging (QPI), and optical diffraction tomography (ODT) similarly enable label-free characterization of cellular morphology, mass, refractive-index distribution, and dynamic changes. Studies have demonstrated detection or characterization of pathogen-associated cellular changes, including bacterial-cell mass and morphology and virus-induced cellular alterations, generally using relatively simple cell-based specimens but with application-dependent sample preparation and image acquisition/analysis requirements [98,99]. These methods can provide rapid measurements once specimens are positioned within the optical system, but their practical turnaround time also depends on image acquisition, computational reconstruction, and classification. Importantly, these approaches primarily measure host–cell or cellular phenotypes unless a pathogen is directly visualized or otherwise characterized and therefore should not be interpreted as equivalent to molecular pathogen detection.

Raman microspectroscopy provides a different label-free strategy by generating molecular vibrational fingerprints that can potentially distinguish microorganisms directly. Recent microfluidic-Raman studies have demonstrated rapid identification and discrimination of bacterial pathogens from small-volume samples, with limited sample preparation compared with conventional culture-based workflows [100,101]. In contrast to OCT and quantitative-phase approaches, Raman measurements can provide direct pathogen-associated molecular phenotyping when the microorganism itself is interrogated. Nevertheless, most reported Raman, QPI, DHM, ODT, and OCT applications remain proof-of-concept or highly application-specific, and evidence for prospectively validated bacterial-versus-viral classification across heterogeneous clinical specimens remains limited. Their potential value therefore lies in providing complementary label-free biological information, particularly where rapid pathogen or cellular phenotyping can be integrated with microfluidics and automated image/spectral analysis, rather than in replacing clinically validated molecular or host-response diagnostics at the present stage of development [102,103].

Unlike pathogen-directed molecular assays and validated host-response classifiers, most current label-free optical and biophysical approaches remain at an application-specific or proof-of-concept stage, and their demonstrated analytical capabilities should not be interpreted as evidence of clinical bacterial-versus-viral discrimination or antimicrobial-stewardship benefit.

The available clinical evidence is also unevenly distributed across infectious syndromes, with a substantial proportion arising from acute respiratory illness, pharyngitis, and sepsis; therefore, evidence from these settings should not automatically be generalized to all infectious diseases, particularly where comparable prospective clinical-utility studies remain limited.

The technologies discussed in this review therefore serve different clinical purposes: pathogen-directed assays identify specific organisms, host-response approaches estimate infectious etiology, hematologic platforms provide complementary physiological information, and sepsis prediction models estimate severity or deterioration risk. Their clinical utility and antimicrobial implications should consequently be evaluated according to their intended use rather than treated as interchangeable bacterial-versus-viral diagnostic approaches.

## 5. Barriers to Clinical Adoption

Despite major advances in rapid diagnostic technologies, their real-world impact on antimicrobial stewardship remains limited by economic, operational, diagnostic, and behavioral barriers. Importantly, these limitations often reflect poor alignment between diagnostic capabilities and the realities of clinical workflows rather than a lack of technological innovation itself [15,21].

One major barrier is the cost and infrastructure required for many advanced diagnostic systems. High-accuracy molecular platforms, including PCR-based assays and syndromic multiplex panels, frequently require expensive instrumentation, trained personnel, and laboratory support [15,16]. These requirements limit implementation in outpatient and urgent-care settings, where a large proportion of unnecessary antibiotic prescribing occurs. Even when available, reimbursement variability and per-test costs may restrict routine utilization.

This access barrier is especially important in low- and middle-income countries, where the burden of AMR is often greatest, yet advanced diagnostic platforms are least available. Technologies such as multiplex PCR systems, host-response immunoassays, transcriptomic classifiers, and AI-enabled hematology platforms often require costly instruments, stable supply chains, trained personnel, and maintenance infrastructure that may be difficult to sustain in resource-limited healthcare systems. As a result, future diagnostic development must prioritize affordable, portable, and simplified platforms that can be deployed in settings where AMR-related morbidity and mortality are most severe.

Importantly, analytical performance alone does not determine the clinical or antimicrobial-stewardship value of a diagnostic technology. A test may demonstrate high accuracy and rapid turnaround yet have limited impact on antibiotic prescribing if its result is unavailable within the relevant clinical decision-making window or does not provide information that directly supports treatment decisions. Therefore, clinical actionability represents a critical dimension of diagnostic utility, linking analytical performance and turnaround time to the ability to inform antibiotic initiation, withholding, escalation, or discontinuation. Technologies that can provide timely and interpretable bacterial-versus-viral information at the point of clinical decision-making may have greater potential to contribute to antimicrobial stewardship, whereas promising technologies without demonstrated effects on prescribing should be considered emerging approaches requiring prospective clinical and implementation evaluation.

Equally important is the persistent mismatch between diagnostic turnaround time and clinical decision-making. In many outpatient and emergency settings, clinicians must determine treatment within minutes of patient evaluation, whereas many “rapid” diagnostics still require 30–60 min or longer to generate results [10,15]. As a result, antibiotics are often prescribed before diagnostic information becomes available, substantially limiting the practical stewardship value of otherwise highly accurate tests.

Diagnostic limitations also contribute to adoption challenges. Rapid antigen tests provide fast results but reduced sensitivity, increasing the risk of false-negative findings [17,37]. Biomarkers such as CRP and procalcitonin provide indirect inflammatory information that must be interpreted within a clinical context [18,51]. Even advanced host-response diagnostics generate probabilistic rather than definitive classifications and may be influenced by disease stage, coinfection, or immune variability [20,66]. These limitations can reduce clinician confidence and discourage reliance on diagnostic outputs during treatment decisions.

Workflow integration represents another major challenge. To influence prescribing behavior, diagnostic tools must fit seamlessly into existing clinical pathways, including sample collection, processing, interpretation, and EHR integration [104,105]. Tests that disrupt workflow or require additional operational steps are less likely to meaningfully alter physician behavior, regardless of analytical performance.

From a biosensor engineering perspective, workflow integration begins before the analytical measurement itself. Sample collection, transport, preparation, target capture, washing, signal generation, and interpretation must be considered as a single system. Microfluidic integration can reduce manual processing and enable automated sample handling, while multiplex sensor architectures can provide complementary pathogen and host-response information from a single specimen. However, these advantages can be offset by matrix-dependent biofouling, nonspecific adsorption, sensor-to-sensor variability, and increased complexity associated with multiplexing. Therefore, device-level performance should ultimately be evaluated according to whether the integrated system can reliably generate interpretable bacterial-versus-viral information within the clinical decision window. This reinforces the broader principle of this review that sample-to-answer integration is not merely an engineering objective; it is a prerequisite for translating biosensor performance into potential antimicrobial-stewardship benefit.

Behavioral and systemic factors further complicate implementation. Diagnostic uncertainty, concern about missing a serious bacterial infection, perceived patient expectations, and clinician risk tolerance all contribute to empiric antibiotic prescribing [11,19]. In addition, limited familiarity with newer technologies and uncertainty regarding interpretation may reduce adoption even when tests are available.

Collectively, these barriers highlight a central problem in the current diagnostic landscape: technologies may demonstrate strong analytical performance yet still fail to influence prescribing behavior if they cannot deliver timely, actionable information within real-world clinical workflows [15,93]. Future diagnostic development must therefore prioritize not only accuracy, but also speed, accessibility, operational simplicity, and workflow compatibility. Emerging host-response and POC hematologic systems may be particularly promising because they are designed to align more closely with the timing and structure of frontline clinical decision-making [20,55].

## 6. Future Directions

The next generation of infectious disease diagnostics will likely be defined less by incremental improvements in analytical accuracy and more by the ability to deliver clinically actionable information within the narrow timeframe of frontline decision-making. Over the next 5–10 years, the field is expected to shift toward integrated diagnostic ecosystems that combine host-response analysis, decentralized POC testing, rapid sequencing technologies, and AI-assisted interpretation into workflow-compatible platforms capable of guiding antimicrobial decisions in real time.

From a technical and translational perspective, future development should move beyond optimization of analytical sensitivity and specificity toward robust sample-to-answer systems that can operate reliably under real-world conditions. Whole-sample processing, including sample preparation, target extraction, reaction, detection, and interpretation, remains an important challenge for decentralized testing because additional manual steps can increase assay time, variability, and operator dependence. For multiplexed platforms, increasing the number of targets also introduces challenges related to cross-reactivity, nonspecific interactions, signal interference, and competition among assay components, requiring rigorous optimization and validation of multiplex performance. Assay reproducibility must likewise be established across operators, instruments, reagent lots, and manufacturing batches. Reagent stability, including preservation of biological recognition elements and other assay components during storage and transportation, is particularly important for POC deployment where continuous cold-chain infrastructure may not be available. In parallel, manufacturability and scalable fabrication should be considered early in platform development to ensure that laboratory-scale performance can be translated into consistent, high-volume production. Standardized calibration procedures, internal controls, and quality-control strategies will be essential for maintaining measurement reliability across devices and clinical sites. Finally, cost-effective translation will require optimization of not only assay reagents but also instrumentation, consumables, sample processing, manufacturing, and operational requirements. Addressing these technical factors alongside clinical validation and workflow integration will be critical for converting promising diagnostic technologies into reproducible, scalable, and clinically actionable tools for antimicrobial stewardship.

### 6.1. Expansion of Host-Response and Immune-Signature Diagnostics

One of the most significant directions in the field is the continued development of host-response diagnostics that evaluate immune activation patterns rather than directly detecting pathogens. Current technologies such as MeMed BV [65,66], FebriDx [70,71], and transcriptomic classifiers [91,92,106] demonstrate the growing feasibility of distinguishing bacterial from viral infection through coordinated immune-response signatures. Over the next decade, these systems may evolve toward larger multi-analyte panels integrating inflammatory proteins, cytokines, transcriptomic signals, and potentially metabolomic biomarkers into unified probabilistic classifiers [20,92].

Future host-response platforms may also incorporate severity prediction and treatment-response monitoring in addition to etiologic classification. Emerging transcriptomic systems such as InSep attempt to classify bacterial likelihood and disease severity using machine-learning analysis of host mRNA expression patterns [92,94]. As sequencing and computational costs continue to decline, transcriptomic profiling may become increasingly feasible in emergency and outpatient settings. However, widespread adoption will depend on reducing assay complexity, improving interpretability, and validating performance across immunocompromised, pediatric, elderly, and co-infected patient populations [92,94].

### 6.2. Decentralized POC and Portable Diagnostic Systems

Another major trend is the continued decentralization of infectious disease diagnostics toward portable POC systems designed for outpatient clinics, urgent-care centers, pharmacies, and resource-limited settings [55,56]. Because antibiotic-prescribing decisions are frequently made within minutes of patient evaluation, technologies capable of delivering rapid results from small sample volumes are likely to exert the greatest real-world stewardship impact [10,15].

Portable hematologic analyzers, compact host-response systems, and fingerstick-based immune-pattern recognition technologies are expected to expand substantially in this area [71,79,84]. These platforms prioritize workflow compatibility, minimal infrastructure requirements, and operational simplicity over maximal analytical complexity. Over time, POC systems may increasingly integrate multiple diagnostic modalities, including CBC-derived immune patterns, inflammatory biomarkers, and rapid molecular detection, into unified portable platforms capable of generating broader diagnostic context during a single patient encounter [55,56].

Low-cost cartridge-based and portable diagnostic systems also represent an important direction for decentralized testing. These technologies aim to simplify complex laboratory workflows into near-patient platforms capable of deployment with minimal technical expertise, particularly in outpatient and resource-limited settings [55,56].

### 6.3. Rapid Sequencing and CRISPR-Based Diagnostics

Rapid sequencing technologies represent one of the most transformative emerging areas in infectious disease diagnostics. Nanopore sequencing platforms, such as those developed by Oxford Nanopore Technologies, enable real-time sequencing of nucleic acids without the need for conventional batch processing [107]. Unlike targeted PCR assays, nanopore systems have the potential to simultaneously identify pathogens, characterize AMR genes, and detect genomic mutations directly from clinical samples within clinically relevant timeframes [107,108].

Although current sequencing workflows remain too complex and resource-intensive for routine outpatient use, continued miniaturization and automation may allow POC sequencing systems to become increasingly practical over the next decade. Whole-genome sequencing (WGS) at the point of care could eventually permit comprehensive pathogen identification and resistance profiling during the initial clinical encounter, fundamentally changing antimicrobial selection strategies [107,108].

CRISPR-based diagnostics represent another rapidly developing technology class. Platforms such as SHERLOCK and DETECTR use CRISPR-associated nucleases to recognize pathogen-specific nucleic acid sequences and generate detectable reporter signals with high specificity [109,110]. Because CRISPR systems combine molecular-level sensitivity with potentially simplified workflows, they may bridge the gap between laboratory-grade molecular diagnostics and decentralized rapid testing. Current research is exploring CRISPR-based approaches for rapid pathogen detection, AMR identification, and multiplex infectious disease testing [109,110].

### 6.4. Artificial Intelligence and Integrated Clinical Decision Support

Artificial intelligence will likely play an increasingly important role in integrating heterogeneous diagnostic data into clinically actionable decision-support systems. Rather than functioning as standalone diagnostics, future AI platforms may synthesize molecular results, host-response biomarkers, CBC parameters, radiographic findings, and physiologic data into unified probabilistic infection models [86,87]. This integration could allow clinicians to move beyond isolated test interpretation toward more comprehensive real-time infection assessment.

AI-assisted diagnostics may also improve antimicrobial stewardship by identifying subtle clinical patterns associated with bacterial disease risk, treatment failure, or deterioration before these patterns become clinically obvious. However, widespread implementation will require rigorous prospective validation, transparency in algorithm development, and careful mitigation of bias and alert fatigue [86,90]. The success of AI systems will depend not only on predictive performance, but also on their ability to integrate seamlessly into clinical workflows without increasing physician burden.

Ultimately, the future of infectious disease diagnostics will depend on achieving alignment between diagnostic timing, workflow integration, and stewardship utility. Technologies that are rapid, minimally invasive, operationally simple, and capable of generating actionable information during the clinical encounter are likely to exert the greatest influence on prescribing behavior [15,93]. Over the next decade, convergence between host-response analysis, portable POC systems, rapid sequencing, and AI-assisted interpretation may substantially reshape the management of infectious disease and provide new tools for addressing the growing global burden of AMR [20,55].

### 6.5. Integrated Biosensor and Microfluidic Sample-to-Answer Platforms

The next stage of biosensor development will increasingly focus on integration rather than isolated improvement of individual sensing components. A clinically useful POC biosensor should ideally combine sample introduction, sample preparation, target enrichment or separation, molecular or affinity recognition, signal transduction, data processing, and result reporting within a single workflow. Microfluidic architectures provide an important technological framework for this integration by enabling controlled transport of small sample volumes, automated reagent delivery, sequential reaction steps, and multiplexed detection within compact cartridges. Recent advances in microfluidic infectious-disease diagnostics demonstrate the potential of these systems to combine sample processing and detection while reducing operator intervention and reagent consumption [111,112].

For bacterial-versus-viral differentiation, future biosensors should move beyond single-analyte detection toward multiplexed pathogen and host-response measurements. A single cartridge could potentially combine bacterial and viral targets with inflammatory biomarkers such as CRP, PCT, or cytokine signatures, allowing direct pathogen evidence and indirect host-response information to be interpreted together. Electrochemical platforms are particularly attractive for this purpose because individually addressable electrodes can support parallel measurements while requiring relatively compact instrumentation. Recent work on electrochemical POC biosensors has emphasized miniaturization, biorecognition strategies, multiplexing, and integration with portable or wireless readout systems [60].

However, achieving sample-to-answer operation remains challenging. Clinical specimens introduce matrix effects and biofouling that can degrade sensor performance, while multiplexing increases the risk of cross-reactivity, signal interference, reagent competition, and calibration drift. Future systems should therefore incorporate antifouling surface engineering, standardized sensor functionalization, integrated sample preparation, quality-control mechanisms, and automated signal normalization. Importantly, device development should be evaluated in clinically relevant matrices and under realistic workflow conditions rather than solely using optimized buffer solutions. This is essential because a biosensor that demonstrates excellent analytical sensitivity but requires extensive sample preparation or produces unstable signals in serum or whole blood may have limited practical value at the point of care [57,58].

Ultimately, the most clinically relevant biosensor platforms will likely be those that combine microfluidic sample handling, robust biorecognition, multiplex transduction, automated signal interpretation, and sample-to-answer operation within a workflow compatible with routine clinical practice. Such integration could allow biosensors to provide complementary pathogen and host-response information within the same clinical encounter, thereby addressing a key limitation of current diagnostic strategies: the separation between analytical sensing and the clinical decision-making process.

## 7. Conclusions

Rapid diagnostic technologies for infectious disease management have advanced substantially over the past decade, encompassing pathogen-directed molecular assays, rapid antigen tests, biomarker platforms, host-response diagnostics, hematologic systems, and emerging AI-assisted decision-support tools. Each approach offers distinct advantages in diagnostic accuracy, speed, accessibility, or clinical interpretability. However, no current modality fully satisfies the combined requirements necessary for optimal antimicrobial stewardship in real-world outpatient and acute-care environments. Molecular systems provide highly accurate pathogen identification but are often constrained by cost, infrastructure, and turnaround time, while faster POC approaches frequently sacrifice analytical precision for workflow compatibility [15,16]. Emerging host-response and transcriptomic platforms represent a particularly promising shift because they address the clinically relevant question of whether antibiotic therapy is warranted rather than simply identifying microbial presence [20,91].

This review highlights that the major limitation of current diagnostic strategies is not insufficient technological capability, but persistent misalignment between diagnostic output and the timing of clinical decision-making. Technologies that generate highly accurate results after prescribing decisions have already been made exert limited practical influence on antibiotic use [15,93]. Consequently, the clinical value of a diagnostic tool is determined not only by analytical performance, but also by its ability to provide timely, interpretable, and actionable information during the patient encounter [10]. From this perspective, future progress in antimicrobial stewardship will likely depend less on maximizing sensitivity alone and more on integrating diagnostic systems into frontline clinical workflows in ways that directly influence physician behavior.

Accordingly, future diagnostic development should prioritize rapid host-response and POC platforms capable of delivering clinically actionable information within minutes, particularly in outpatient and urgent-care settings. However, analytical performance and rapid turnaround should not be considered sufficient evidence of antimicrobial-stewardship benefit. Future research should prioritize prospective clinical and pragmatic randomized studies evaluating whether diagnostic results alter antibiotic initiation, discontinuation, total treatment duration, time to appropriate therapy, reconsultation, hospitalization, adverse events, and mortality. Such studies should also evaluate whether diagnostic results are integrated into explicit clinical decision pathways, because testing alone may not reliably change prescribing behavior.

## Data Availability

No new data were created or analyzed in this study. Data sharing is not applicable to this article.
